# Supplementary figures and images for: Auditory cortex neurons that encode negative prediction errors respond to omissions of sounds in a predictable sequence
Source: PLoS Biol. 2025 Jun 18;23(6):e3003242. doi: 10.1371/journal.pbio.3003242 (PMC12212881; doi:10.1371/journal.pbio.3003242)

A

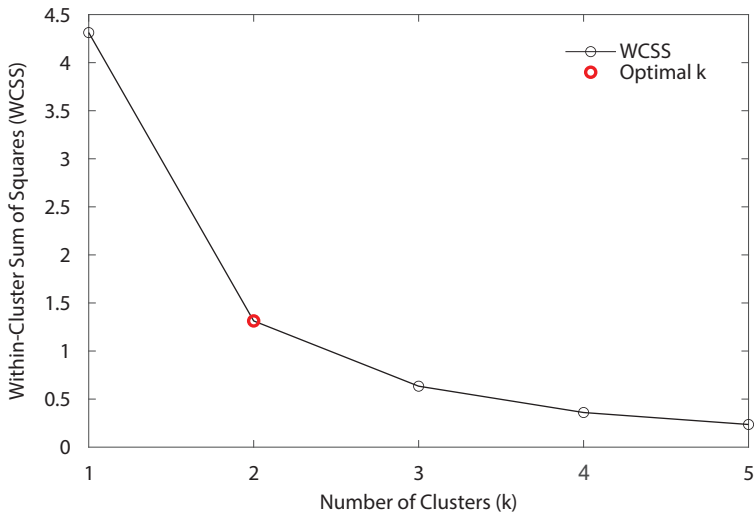

Supplement: S1 Fig — (A) The elbow method was used to determine the optimal number of clusters for classifying neurons based on the correlation between omission response and OP tone probability. The x-axis represents the number of clusters, while the y-axis represents the WCSS, which measures clustering compactness. A sharp inflection point, or “elbow,” is observed at k = 2, indicating that a two-cluster solution best balances model complexity and explained variance. This supports the classification of neurons into two distinct response groups. (PDF) [file pbio.3003242.s001.pdf]

A

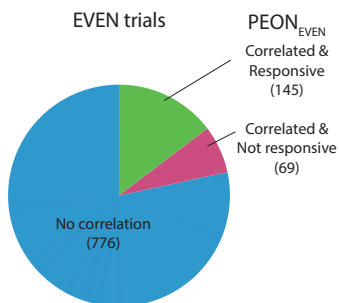PEON<sub>EVEN</sub> on ODD trials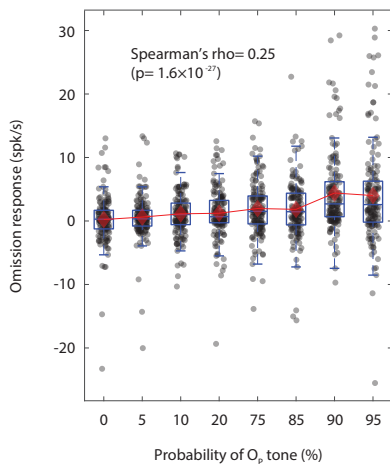

B

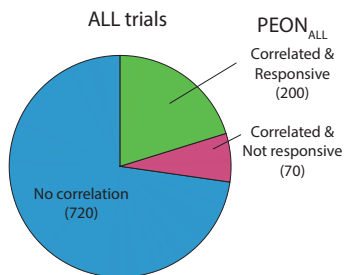PEON<sub>ALL</sub> on ALL trials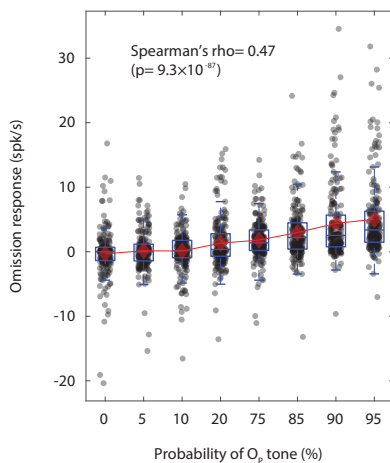

Supplement: S2 Fig — (A) left: Classification of PEONEVEN. Pie chart, illustrating how neurons identified in odd trials were categorized when evaluated on even trials. Neurons are grouped based on whether they exhibit no correlation with tone probability, correlation but no omission response, or both correlation and a measurable omission response (“PEONEVEN”). Underlying data are provided in S3 Data (sheet “S2A_Individual_Points”). (A) right: Box plot summarizing omission responses for PEONEVEN, tested on ODD trials. The x-axis represents the OP tone probability (0%–95%), and the y-axis indicates mean baseline-subtracted firing rate in response to the omission, calculated over a 5–120 ms window after the expected tone onset. Boxes correspond to the IQR with a horizontal line marking the median. Whiskers extend to 1.5× IQR. Individual data points are shown as overlaid dots, with means at each probability level marked by red diamonds connected by a dashed line. The text annotation shows the Spearman’s rank correlation coefficient (rho) and associated p-value, quantifying the strength and statistical significance of the monotonic relationship between probability and neural response. (B) left: Pie chart showing neuron categorization when using all available trials without splitting into odd or even subsets. Underlying data are provided in S3 Data (sheet “S2B_Individual_Points”) (B) right: Box plot displaying omission responses for PEONs identified using all trials, allowing direct comparison with split-data analyses. (PDF) [file pbio.3003242.s002.pdf]

A

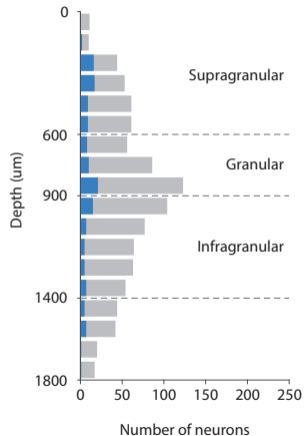

B

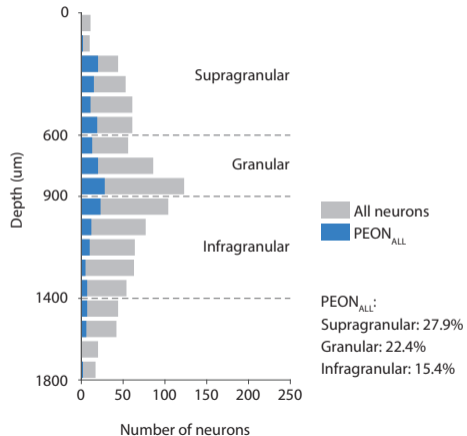

Supplement: S3 Fig — (A) Laminar distribution of PEONEVEN. The distribution of the recorded population (gray bars) and the neurons classified as PEON (blue bars) is shown as a function of cortical depth (y-axis). Horizontal dashed lines indicate the approximate boundaries between supragranular (0–600 μm), granular (600–900 μm), and infragranular (900–1,400 μm) layers, based on current source density analysis. (B) Laminar distribution of PEONALL. Same as (A), but for PEONALL, showing the depth distribution when using all available trials without splitting into odd and even subsets. (PDF) [file pbio.3003242.s003.pdf]

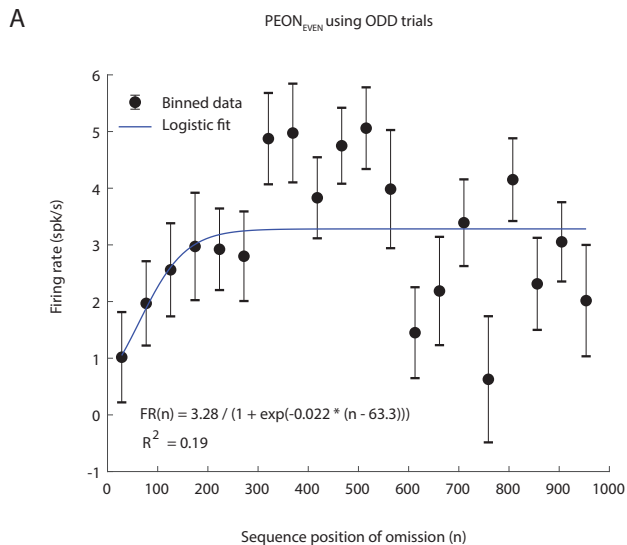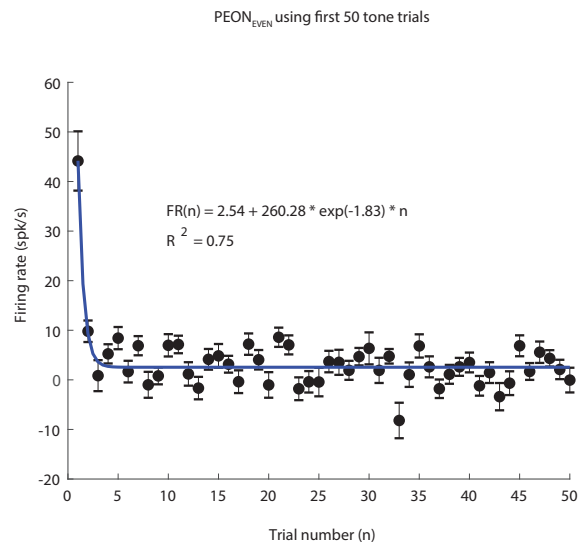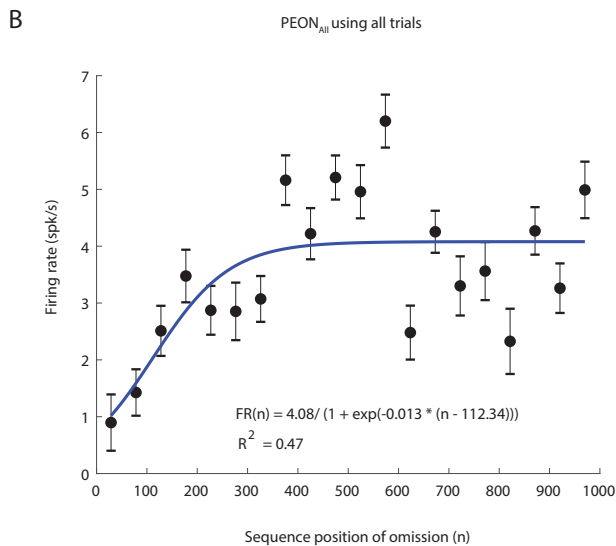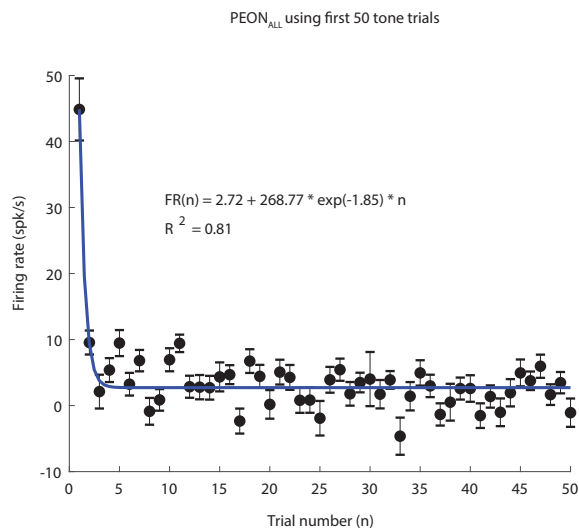

Supplement: S4 Fig — (A) Left: Firing rate of PEONEVEN for omission events (on ODD trials) in the four conditions where the OP tone was the standard (75%, 85%, 90%, 95%), grouped by sequence position. The x-axis represents the bin center (i.e., sequence position), and the y-axis represents the firing rate in spikes per second. The black dots indicate the mean firing rate across all PEONEVEN for each bin, and the error bars represent SEM, showing variability across neurons. The blue line represents the logistic growth curve fit to the data, illustrating how omission responses evolve over successive standards. Right: Firing rate of PEONEVEN in response to the presentation of tones across the first 50 trials. The x-axis represents the trial number, and the y-axis represents the firing rate in spikes per second. The black dots indicate the mean firing rate across all PEONEVEN for each trial, and the error bars represent SEM, showing variability across neurons. The blue line represents the exponential decay model fit to the data, indicating a rapid decline in response intensity over the initial trials as the neurons adapt to the repeated tone presentations. (B) Left: Firing rate of PEONALL for omission events. Same as (A, left), but for PEONALL, showing the omission response buildup when using all available trials without splitting into odd and even subsets. Right: Firing rate of PEONALL in response to tone presentations. Same as (A, right), but for PEONALL, showing the adaptation to standard tones across the first 50 trials using all available trials. (PDF) [file pbio.3003242.s004.pdf]

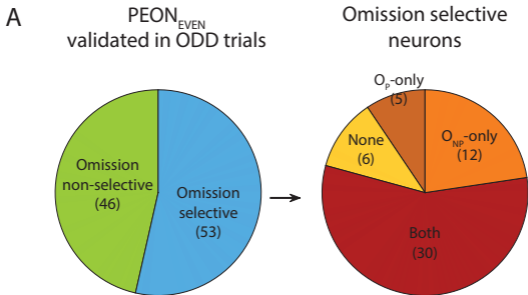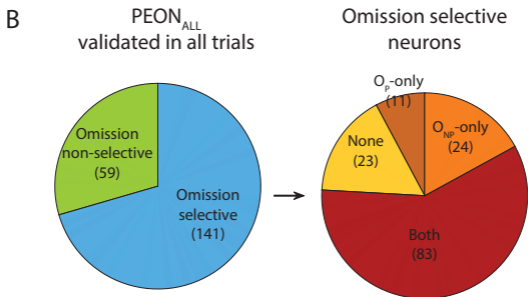

Supplement: S5 Fig — (A) Left: Pie chart categorizing 99 PEONEVEN neurons (identified on even trials and validated on odd trials) into omission non-selective (46) and omission selective (53). Right: Among the 53 omission-selective neurons, 5 responded exclusively to the OP tone (OP-only), 12 responded exclusively to the ONP tone (ONP-only), 6 did not significantly respond to either tone (None), and 30 responded to both tones (Both). (B) Left: Pie chart categorizing 200 PEONALL neurons into omission non-selective (59) and omission selective (141). Right: Among the 141 omission-selective neurons, 11 responded exclusively to OP (OP-only), 24 to ONP (ONP-only), 23 to neither tone (None), and 83 to both (Both). (PDF) [file pbio.3003242.s005.pdf]

**A**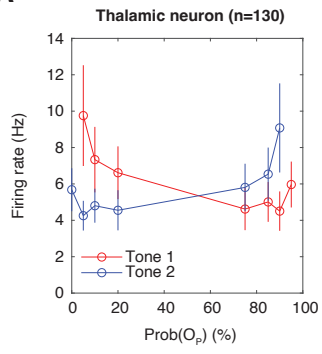**B**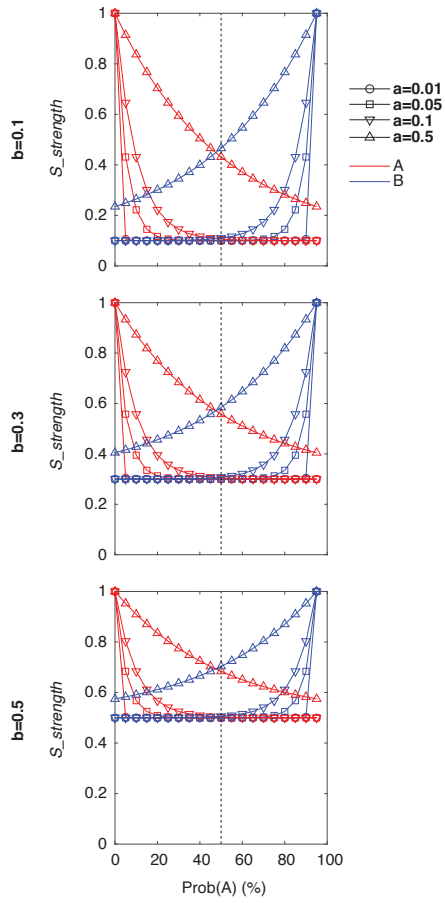**C**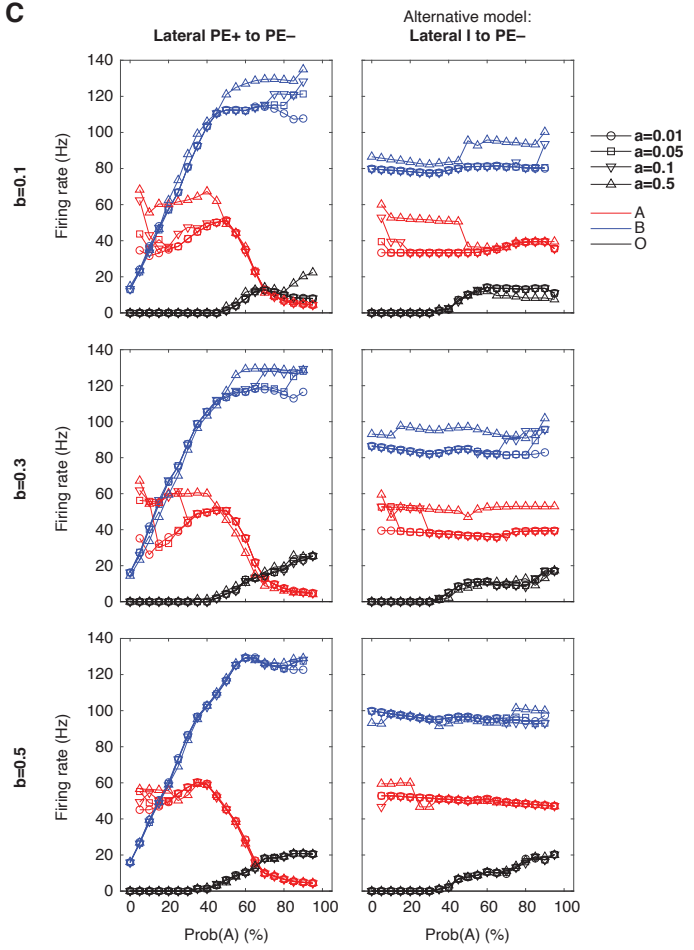

Supplement: S6 Fig — (A) Firing rates of thalamic neurons (n = 130) for Tone 1 (red) and Tone 2 (blue) across varying probabilities of the preferred tone (Prob(OP)). Error bars indicate SEM. (B) To mimic the thalamic sensory inputs shown in the panel A, the strength of sensory signals (S_strength) for Tones A (red) and B (blue) was modelled as exponential decay functions. Simulations with various combinations of the adaptation factor a and base firing factor b were performed, and the results are shown with different symbols. (C) Comparison of mean firing rates in different model configurations: Lateral PE+ to PE– (left), Lateral I to PE– (middle), and No lateral (right). Firing rates for Tones A (red), B (blue), and omissions (O, black) are plotted against Prob(A). The same symbols are used as in panel B. (PDF) [file pbio.3003242.s006.pdf]

- Omission response
- Tone response to  $O_p$
- ◆ Tone response to  $O_{NP}$

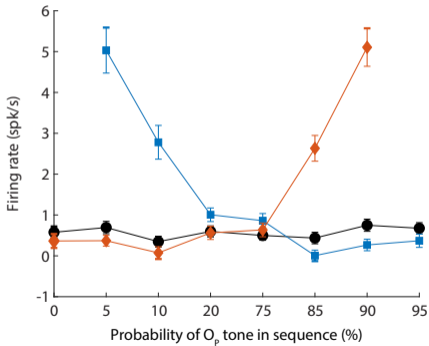

Supplement: S7 Fig — (A) Population-level firing rates comparing omission responses (black), tone responses to the OP tone (blue), and tone responses to the ONP tone (red), plotted against the probability of the OP tone. The y-axis indicates firing rate (spikes/s), and error bars show standard error of the mean. All data are drawn from the EVEN trials of non-PEONs identified in ODD trials. (PDF) [file pbio.3003242.s007.pdf]

A

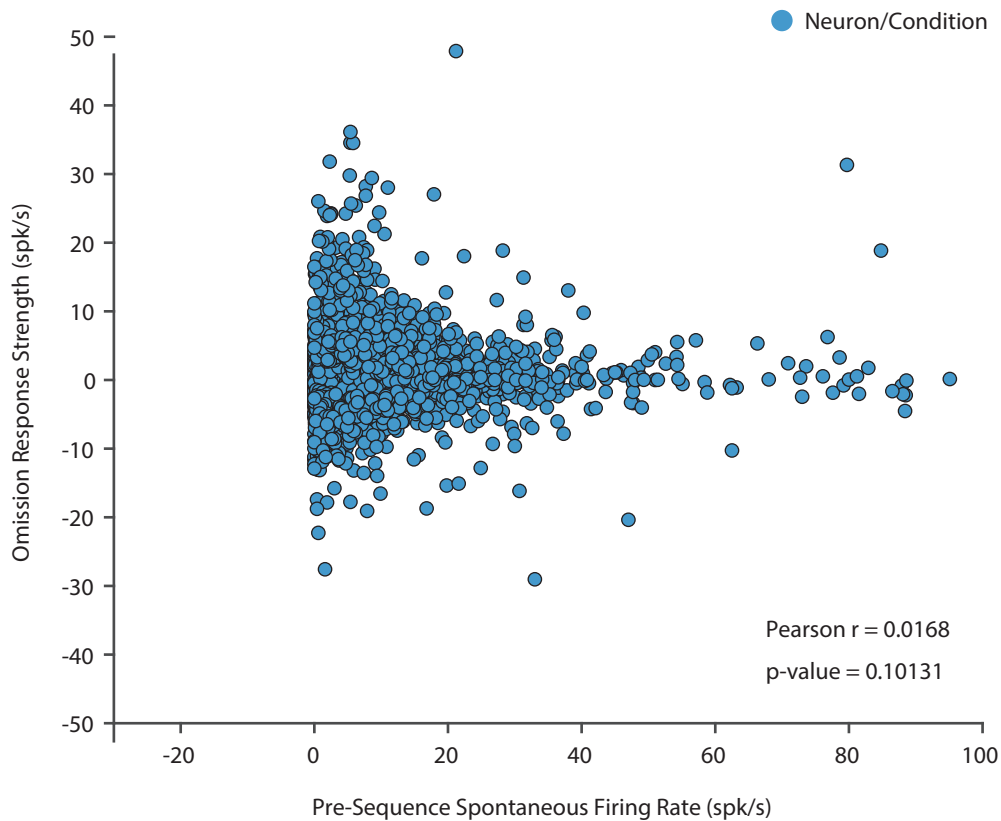

Supplement: S8 Fig — (A) Scatterplot of pre-sequence spontaneous firing rate versus omission response strength. Each point represents a single neuron in a specific probability condition. The x-axis shows the pre-sequence spontaneous firing rate (spikes/s), measured before the onset of tone sequences, while the y-axis indicates the omission response strength (spikes/s). The Pearson correlation coefficient (r) and p-value are displayed in the lower-right corner. Underlying data are provided in S4 Data. (PDF) [file pbio.3003242.s008.pdf]
