# Supplementary material for: Auditory cortex neurons that encode negative prediction errors respond to omissions of sounds in a predictable sequence
Source: PLoS Biol. 2025 Jun 18;23(6):e3003242. doi: 10.1371/journal.pbio.3003242 (PMC12212881; doi:10.1371/journal.pbio.3003242)
Supplement: S1 Text — This supplement provides the mathematical framework discussed in the main text under "Lateral prediction suppression and the free energy principle." It formally derives how suppressive lateral interactions between sensory streams are a natural consequence of applying the free energy principle to a simplified auditory model. (DOCX) [file pbio.3003242.s013.docx]

# Supplemental Information

**Simplified Free Energy Principle:**

First, we assumed that the amplitudes of the sensory input (auditory tone) at the i-th frequency (u_i_) are generated by a single-hierarchy static linear generative model:

$u_{i}=\theta_{i}v_{i}+\varepsilon_{ui}$

$v_{i}=v_{pi}+\varepsilon_{pi}$

(Eq. 1)

where v_i_ is a hidden variable to generate auditory tones at the i-th frequency, θ_i_ is a linear gain between v_ii_ and u_i_, and ε_ui_ and ε_pi_ are Gaussian noise. We assumed that the linear gain θ_i_, the prior expectation of the hidden variables $v_{pi}$, and the covariance matrices $\Sigma_{u}$, $\Sigma_{p}$ are already adapted to the natural environment and stable over time, at least on the time scale of the electrophysiological experiments.

We assumed only one hierarchy in the predictive coding network because the generative model also has only one hierarchy. The estimate of the hidden variable at the i-th frequency ($v_{i}$) is denoted as $\phi_{i}$.

According to Friston[65], hidden variables φ_I_ can be estimated by:

$$\frac{d\phi_{i}}{dt}=\Sigma_{j}\theta_{i}\left( \Sigma_{u}^{-1} \right)_{ij}\left( u_{j}-\theta_{j}\phi_{j} \right)-\Sigma_{j}\left( \Sigma_{p}^{-1} \right)_{ij}\left( \phi_{j}-V_{pj} \right)$$

 (Eq. 2)

We defined signal, prediction, bias, and prediction error as follows:

$$S_{i}\left( t \right)=u_{i}$$

$$P_{i}\left( t \right)=\theta_{i}\phi_{i}$$

$$V_{i}=\theta_{i}v_{pi}$$

$$E_{i}\left( t \right)=S_{i}\left( t \right)-P_{i}\left( t \right)$$

(Eq. 3)

S_i_(t) is the sensory (auditory tone) amplitude at the i-th frequency, and P_i_(t)_i_ is the prediction of the tone amplitude at the i-th frequency. V_i_ is the bias due to the prior expectation of the tone amplitude at the i-th frequency. E_i_(t) is the prediction error. We assumed that S_i_(t) is always positive or zero, because it represents the amplitude of the tone. The prediction error can be positive or negative and we separated the positive and negative prediction errors as follows: $Ep_{i}\left( t \right) = max(0, E_{i}\left( t \right)) (positive error)$

$En_{i}\left( t \right) = max(0, {-E}_{i}\left( t \right)) (negative error)$

(Eq. 4)

Then, Eq. 2 can be rewritten as follows:

$$\frac{dP_{j}\left( t \right)}{dt}=\Sigma_{j}\theta_{i}\theta_{i}\left( \Sigma_{u}^{-1} \right)_{ij}\left( S_{j}\left( t \right)-P_{j}\left( t \right) \right)-\Sigma_{j}\left( \frac{\theta_{i}}{\theta_{j}} \right)\left( \Sigma_{p}^{-1} \right)_{ij}\left( P_{j}\left( t \right)-V_{j} \right)$$

$$=\Sigma_{j}\theta_{i}\theta_{i}\left( \Sigma_{u}^{-1} \right)_{ij}\left( Ep_{j}\left( t \right)-En_{j}\left( t \right) \right)-\Sigma_{j}\left( \frac{\theta_{i}}{\theta_{j}} \right)\left( \Sigma_{p}^{-1} \right)_{ij}\left( P_{j}\left( t \right)-V_{j} \right)$$

(Eq. 5)

We assumed the correlation structures of ε_ui_ and ε_pi_ as follows:

$\left( \Sigma_{u} \right)_{ii}=\sigma_{ui}^{2}$ (variance)

$\left( \Sigma_{u} \right)_{ii}\left( i\neq j \right)=\sigma_{ui}\sigma_{uj}r_{uij}$ (covariance)

$\left( \Sigma_{p} \right)_{ii}=\sigma_{pi}^{2}$ (variance)

$\left( \Sigma_{p} \right)_{ii}\left( i\neq j \right)=\sigma_{pi}\sigma_{pj}r_{pij}$ (covariance)

(Eq. 6)

where r_uij_ is a correlation coefficient between i-th frequency and j-th frequency.

When we considered only two tone frequencies, similar to the electrophysiological experiments, we can obtain the precision matrices (Σ_u_^-1^)_ij_ and (Σ_p_^-1^)_ij_, that are the inverse of the covariance matrices as follows:

$\Delta=\Sigma_{u}\left( S_{u} \right)=\sigma_{u1}^{2}\sigma_{u2}^{2}-\left( \sigma_{u1}\sigma_{u2}r_{u12} \right)^{2}=\sigma_{u1}^{2}\sigma_{u2}^{2}\left( 1-r_{u12}^{2} \right)$

$\left( {\Sigma_{u}}^{-1} \right)_{11}=\frac{\left( \Sigma_{u} \right)_{22}}{\Delta}=\frac{\sigma_{u2}^{2}}{\sigma_{u1}^{2}\sigma_{u2}^{2}\left( 1-{r_{u12}}^{2} \right)}=\frac{1}{\sigma_{u1}^{2}\left( 1-r_{u12}^{2} \right)}=k_{u1}^{2}$

$\left( {\Sigma_{u}}^{-1} \right)_{22}=\frac{\left( \Sigma_{u} \right)_{11}}{\Delta}=\frac{\sigma_{u1}^{2}}{\sigma_{u1}^{2}\sigma_{u2}^{2}\left( 1-{r_{u12}}^{2} \right)}=\frac{1}{\sigma_{u2}^{2}\left( 1-r_{u12}^{2} \right)}=k_{u2}^{2}$

$\left( {\Sigma_{u}}^{-1} \right)_{12}=\left( {\Sigma_{u}}^{-1} \right)_{21}=\frac{-\left( \Sigma_{u} \right)_{12}}{\Delta}=\frac{-\sigma_{u1}\sigma_{u2}r_{u12}}{\sigma_{u1}^{2}\sigma_{u2}^{2}\left( 1-r_{u12}^{2} \right)}=\frac{-r_{u12}}{\sigma_{u1}\sigma_{u2}\left( 1-r_{u12}^{2} \right)}=-k_{u1}k_{u2}r_{u12}$

where we defined

$k_{u1}^{2}=\frac{1}{\sigma_{u1}\sqrt{\left( 1-r_{u12}^{2} \right)}}>0$

$k_{u2}^{2}=\frac{1}{\sigma_{u2}\sqrt{\left( 1-r_{u12}^{2} \right)}}>0$

(Eq. 7)

Similarly,

(Σ_p_^-1^)_11_ = $k_{p1}^{2}$

(Σ_p_^-1^)_22_ = $k_{p2}^{2}$

(Σ_p_^-1^)_12_ = (Σ_p_^-1^)_21_ = $-k_{p1}k_{p2}r_{p12}$

where we defined

${k_{p1}}^{2}=\frac{1}{\sigma_{p1}\sqrt{\left( 1-r_{p12}^{2} \right)}}>0$

${k_{p2}}^{2}=\frac{1}{\sigma_{p2}\sqrt{\left( 1-r_{p12}^{2} \right)}}>0$

(Eq. 8)

Then, Eq. 5 can be rewritten as follows:

$\frac{dP_{1}\left( t \right)}{dt}=\theta_{1}^{2}\left( \Sigma_{u}^{-1} \right)_{11}\left( Ep_{1}\left( t \right)-En_{1}\left( t \right) \right)+\theta_{1}\theta_{2}\left( \Sigma_{u}^{-1} \right)_{12}\left( Ep_{2}\left( t \right)-En_{2}\left( t \right) \right)$ $-\left( \Sigma_{p}^{-1} \right)_{11}\left( P_{1}\left( t \right)-V_{1} \right)-\left( \frac{\theta_{1}}{\theta_{2}} \right)\left( \Sigma_{p}^{-1} \right)_{12}\left( P_{2}\left( t \right)-V_{2} \right)$ $=\theta_{1}^{2} k_{u1}^{2}\left( Ep_{1}\left( t \right)-En_{1}\left( t \right) \right)-\theta_{1}\theta_{2}k_{u1}k_{u2}r_{u12}\left( Ep_{2}\left( t \right)-En_{2}\left( t \right) \right)$ $-k_{p1}^{2}\left( P_{1}\left( t \right)-V_{1} \right)+\left( \frac{\theta_{1}}{\theta_{2}} \right)k_{p1}k_{p2}r_{p12}\left( P_{2}\left( t \right)-V_{2} \right)$

$\frac{dP_{2}\left( t \right)}{dt}=\theta_{2}^{2}\left( \Sigma_{u}^{-1} \right)_{22}\left( Ep_{2}\left( t \right)-En_{2}\left( t \right) \right)+\theta_{1}\theta_{2}\left( \Sigma_{u}^{-1} \right)_{21}\left( Ep_{1}\left( t \right)-En_{1}\left( t \right) \right)$ $-\left( \Sigma_{p}^{-1} \right)_{22}\left( P_{2}\left( t \right)-V_{2} \right)-\left( \frac{\theta_{2}}{\theta_{1}} \right)\left( \Sigma_{p}^{-1} \right)_{21}\left( P_{1}\left( t \right)-V_{1} \right)$ $=\theta_{2}^{2} k_{u2}^{2}\left( Ep_{2}\left( t \right)-En_{2}\left( t \right) \right)-\theta_{1}\theta_{2}k_{u1}k_{u2}r_{u12}\left( Ep_{1}\left( t \right)-En_{1}\left( t \right) \right)$ $-k_{p2}^{2}\left( P_{2}\left( t \right)-V_{2} \right)+\left( \frac{\theta_{2}}{\theta_{1}} \right)k_{p1}k_{p2}r_{p12}\left( P_{1}\left( t \right)-V_{1} \right)$

(Eq. 9)

Further, we assumed that the noises of the hidden variables (ε_pi_) are independent over different frequencies:

$r_{p12}=0$

(Eq. 10)

Then, we finally obtained the prediction update rule consisting of three terms:

$\frac{dP_{1}\left( t \right)}{dt}=\theta_{1}^{2} k_{u1}^{2}\left( Ep_{1}\left( t \right)-En_{1}\left( t \right) \right)-\theta_{1}\theta_{2}k_{u1}k_{u2}r_{u12}\left( Ep_{2}\left( t \right)-En_{2}\left( t \right) \right)-k_{p1}^{2}\left( P_{1}\left( t \right)-V_{1} \right)$

$\frac{dP_{2}\left( t \right)}{dt}=\theta_{2}^{2} k_{u2}^{2}\left( Ep_{2}\left( t \right)-En_{2}\left( t \right) \right)-\theta_{1}\theta_{2}k_{u1}k_{u2}r_{u12}\left( Ep_{1}\left( t \right)-En_{1}\left( t \right) \right)-k_{p2}^{2}\left( P_{2}\left( t \right)-V_{2} \right)$

(Eq. 11)

The first term, θ_1_^2^ k_u1_^2^ (Ep_1_(t) - En_1_(t)), represents the update by the positive and negative prediction errors in the same stream. The second term, – θ_1_ θ_2_ k_u1_ k_u2_ r_u12_ (Ep_2_(t) - En_2_(t)), represents the lateral interaction of positive and negative prediction errors across steams of the 1^st^ and 2^nd^ frequencies. The third term, – k_p1_^2^ (P_1_(t) –V_1_), represents the temporal decay of the prediction towards the prior expectation.

Thus, the lateral interaction of positive and negative errors across different streams is a natural outcome derived directly from the free energy principle. The sign of the lateral interaction depends on the correlation between the noise of the sensory input (ε_ui_) at the 1^st^ and 2^nd^ frequencies. In general, the amplitudes of tones at two different frequencies are positively correlated in the natural environment [56], the lateral interaction becomes suppressive.
