# Supplementary material for: Auditory cortex neurons that encode negative prediction errors respond to omissions of sounds in a predictable sequence
Source: PLoS Biol. 2025 Jun 18;23(6):e3003242. doi: 10.1371/journal.pbio.3003242 (PMC12212881; doi:10.1371/journal.pbio.3003242)
Supplement: S1 Table — This table contains the core parameters for the intra-stream connections of the neural circuit model (5A Fig). (DOCX) [file pbio.3003242.s014.docx]

# Supplemental Table

**Table S1. Synaptic delays and weights within each stream module.**

| **Connection** | **Delay** | **Weight** |
| --- | --- | --- |
| I to I– | 10 ms | 0.5 µA |
| I to PE+ | 20 ms | 0.5 µA |
| P to PE– | 20 ms | 0.5 µA |
| P to I+ | 10 ms | 0.5 µA |
| I– to PE– | 5 ms | 0.2 µA |
| I+ to PE+ | 5 ms | 0.2 µA |
