# Supplementary material for: Auditory cortex neurons that encode negative prediction errors respond to omissions of sounds in a predictable sequence
Source: PLoS Biol. 2025 Jun 18;23(6):e3003242. doi: 10.1371/journal.pbio.3003242 (PMC12212881; doi:10.1371/journal.pbio.3003242)
Supplement: Attachment — Submitted filename: PLOS Biology Response Letter 031125AY.pdf [file pbio.3003242.s015.pdf]

REVIEWER REPORTS: PBIOLOGY-D-24-02638R1

Reviewer #1

Review of "omission-responsive neurons encode negative prediction error and probability in the auditory cortex"

> *In their manuscript entitled "Omission-responsive neurons encode negative prediction error and probability in the auditory cortex", Yaron et al. revisit neural responses in the auditory cortex to omissions of sounds in a predictable sequence, with an interesting extension in the classical paradigm, namely considering a range of different probabilities in the conditioning sequence, which offers them a few interesting possibilities in the analysis: (1) compare whether omission responses in certain neurons are sensitive to the probability of the sound in the sequence, (2) compare whether omission responses in certain neurons depend on the (frequency of the) stimulus, and (3) provide additional constraints for the comparison of different models. In particular from the latter point, they converge to predicting a new model, and relate this to the free-energy principle. While I think this overall provides interesting new findings, I think the rationale of the manuscript is too focussed on proving a single point, while ignoring alternatives and providing appropriate controls, which I spell out in detail below in the major points.*

We thank the reviewer for understanding the key points of our paper, and appreciate the critical feedback.

> *1.1. The way the analysis is run now is susceptible to selection bias, i.e. the whole data set is used for determining whether a cell shows a significant omission response, and then the same data is averaged to determine the effect size. To get a more truthful estimate of effect size, i.e. Omission response, it is essential to perform split-data analysis, i.e. determine significant cells first on half of the data (randomly selected omissions), and then average the other half for display and further analysis of the response. As I understand that this creates a significant burden in terms of rewriting analysis, I would suggest to limit this to Fig. 2D, in particular to see whether the difference between the Non-ORN and ORN cells shrinks.*

We thank the reviewer for raising this critical issue regarding potential selection bias in our original analysis, particularly in relation to the initial classification of neurons as Omission-responsive neurons (ORNs). We recognize that using the entire dataset both to identify neurons with significant omission responses and to characterize their properties could lead to an overestimation of effect sizes due to circularity.

To address this concern, we have fundamentally revised our analysis in two key ways. First, we have eliminated the preliminary ORN/non-ORN classification as well as the classification of "auditory neurons". We now directly identify Probability-Encoding Omission Neurons (PEONs) based on their probability-dependent responses to omissions. This approach avoids the selection bias inherent in the original ORN classification, as it is

based on relationships across conditions rather than on significant responses irrespective of probability.

Second, as you suggested, we have implemented a robust split-half analysis to ensure that neuron selection and characterization are performed on independent data. In this revised approach, we divide trials into two independent subsets—ODD and EVEN trials. One subset (the training set) is used to identify PEONs based on two criteria: a significant correlation between omission responses and the probability of the "omission preferred" (O_P), and a significant positive omission response in conditions where the O_P tone is the standard and thus expected to yield large responses. The other subset (the testing set) is then used to characterize the response properties of the identified PEONs. This process is repeated with the roles of ODD and EVEN trials swapped, ensuring that selection and characterization remain independent and avoiding circularity. Additionally, we have included an analysis using all trials to provide a comprehensive picture.
While this refinement led to changes in the specific numbers of neurons classified as PEONs and in some reported statistical values, it did not alter the overall conclusions of the study. The core findings regarding PEONs and their probability encoding remain robust.

Below, we provide a detailed account of the specific changes made:
In the Methods section, we added a section on the split-half analysis:

> "To mitigate potential biases such as double dipping—where the same data are used both to select and evaluate effects—and to ensure the robustness of our probability encoding analysis, we employed a split-half approach. For each probability condition, the 50 omission trials were divided into two equal halves—an odd-indexed "training set" and an even-indexed "testing set," with each set providing 25 omission trials per condition. Using only the training set, we computed Spearman's rank correlation between each neuron's omission response and the probability condition. neurons with a significant correlation ($p < 0.05$) were provisionally labeled as "correlated," and the O_P tone for each neuron was defined based on the direction of the correlation in the test data. From this subset of correlated neurons, we then pooled the training-set trials in the conditions where the O_P was standard (75–95%) and applied a one-sample Wilcoxon sign-rank test ($p < 0.05$) to determine whether their omission response was significantly above zero. Because the omission response is computed as the firing rate minus a local baseline, testing against zero effectively assesses whether post-omission activity exceeds the neuron's immediate pre-omission baseline. Only neurons that exhibited both a significant correlation and a robust omission response were designated as PEONs in the training set. All subsequent population plots—such as boxplots or group correlations—were generated solely from the testing set, ensuring that any observed trends were unbiased by the training-set selection process. Complementary analyses were also carried out using even-indexed trials for training (with odd-indexed trials for testing) as well as a non-split method utilizing all trials; these additional analyses, presented in the Supplementary Materials, confirm that our main conclusions do not depend on the specific trial split." (page 36 line 857 - page 37 line 877)

This change resulted in an update to Figure 2. The new split analysis is now reflected on the figure and corresponding text in the results section:

> “To further examine these PEONs without introducing double-dipping biases, we conducted a split-data analysis by dividing trials into two independent subsets (odd and even trials, denoted as ODD and EVEN trials) to ensure the robustness of our analyses. Using the ODD trials, we identified 123 PEONs according to two criteria: (1) a significant correlation ($p < 0.05$) between their omission responses and the probability of the corresponding O_P tone, as verified by Spearman’s correlation test; and (2) a significant omission response ($p < 0.05$, Wilcoxon sign-rank test) to this O_P tone in the four conditions where it was presented as standard (75%, 85%, 90%, and 95%), when the O_P tone is most predictable and omission responses should be greatest (see details in Methods). We refer to these PEONs as $PEON_{ODD}$. To confirm the reliability of our selection criteria and ensure that the identified neurons were not specific to one trial subset, we applied the same classification procedure to the EVEN trials, identifying a separate group of PEONs referred to as $PEON_{EVEN}$. For additional comparison, maximizing statistical power and evaluating probability encoding without trial-based data splitting, we identified $PEON_{ALL}$, a group classified using all available trials.”(page 8 line 159 - page 9 line 172)

Furthermore, the split-half analysis was incorporated into Figures 3, 4, Supplementary Figures S2-5, and their corresponding text, to ensure consistency and rigor across all analyses involving PEONs.

1.2. The firing rate of the neurons shown in Fig. 2D dips below 0, indicating that it has been normalized, however, as far as I could see, it is not specified how. In addition to adding this information, it would be important to show the neurons' pre-sequence spontaneous rates in all panels of Fig. 2, to get a sense of whether the Omission responses are merely returning to baseline rate, or a genuine increase in response.

We thank the reviewer for pointing out the lack of clarity in our description of the firing rate calculation and for raising the important question of whether omission responses represent a genuine increase in activity or simply a return to baseline. We realize that we should have been more clear about how the firing rates were calculated.

In the revised manuscript, we have clarified the firing rate calculation in both the methods section and the relevant result.While Figure 2D is no longer relevant because we no longer use the ORN categorization, the firing rates plotted in our new figures such as 2B (and throughout the manuscript, where applicable) represent baseline-subtracted firing rates. The negative values observed in some cases reflect instances where the firing rate in the omission window was lower than the firing rate in the immediately preceding baseline window. Furthermore, we performed a new analysis to examine the pre-sequence spontaneous firing rates. This analysis, detailed in the Supplementary Materials, revealed no correlation between pre-sequence spontaneous rates and omission responses. This lack of correlation argues against a simple return to a pre-sequence baseline.

In the Results section, we added the following:

> “The omission response was defined as the neuron’s mean firing rate during a 5 to 120 ms window after the omission, with a local baseline subtracted on a per-trial basis. This baseline was calculated as the mean firing rate from –24 to 5 ms relative to the expected tone onset, capturing the neuron’s state just before the tone was omitted [26]” (page 7 lines 131-134)

In the Methods section, we added the following:

> "Omission response was defined as the firing rate measured in a 5–120 ms post-omission window, with a local baseline subtracted on a per-trial basis. The baseline was computed as the mean firing rate within a −24 ms to +5 ms window relative to the expected tone onset, a period that closely reflects the neuron's state immediately prior to the omitted tone and controls for transient adaptations or fluctuations that might occur mid-sequence. The computed baseline was then subtracted from the mean firing rate obtained during the post-omission period, yielding a final omission response value that was typically around zero when the neuron exhibited no net change in firing rate. Although we corrected responses using this immediate pre-omission baseline, we also examined pre-sequence spontaneous firing rates and observed no correlation between those rates and omission responses (see Supplementary Materials, Figure S8). This methodology is consistent with established work [26], which demonstrated robust omission responses using a similar immediate reference period." (page 35 line 829 - page 36 line 840)

A

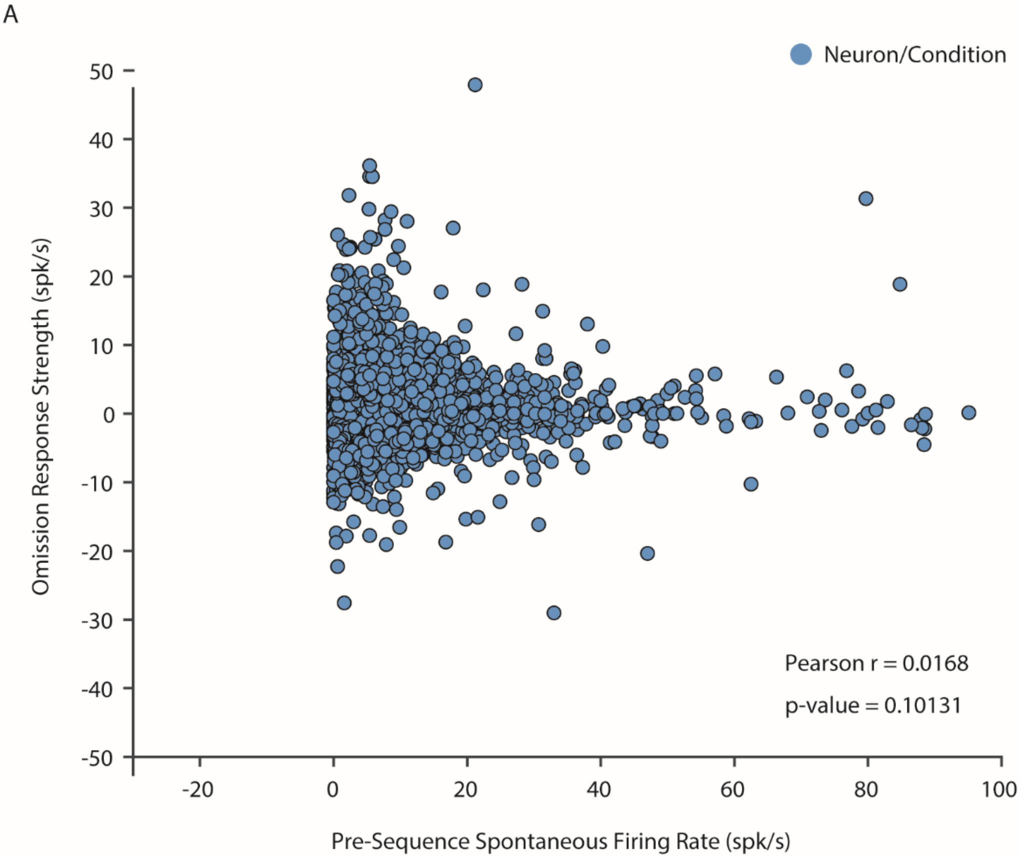

Moreover, a 'return to baseline' interpretation is inconsistent with several key findings of our study. PEONs exhibit omission responses that are not only selective—scaling strongly with the probability of one of the tones—but also build up gradually over successive trials with the same expected tone (Figure 3A). A fixed baseline return would predict a consistent omission response magnitude, not a progressive increase reflecting the gradual strengthening of predictions and the resulting larger prediction error when an omission occurs. Additionally, if omission selectivity simply reflected pre-existing tone preferences or local baseline shifts due to short-term adaptation, we would expect a positive correlation between a neuron's preference for a presented tone and its preference for the omission of that tone. However, we found no such relationship (see response to comment 1.6)

Together, these findings suggest that the omission responses, particularly in PEONs, represent a genuine, context-dependent increase in firing rate—consistent with negative prediction error signaling—rather than merely a return to a pre-defined baseline firing rate.

1.3 Relating to the difference between Non-ORNs and ORNs, it would be important to show whether their Omission responses form a continuum or two clusters/groups, i.e. in addition to the averages also show distributions of the omission response of all cells, and the Non-ORNs and the ORNs side-by-side. If the responses form more of a continuum, this would call the binary differentiation between these cell types into question, which is used in the model.

We thank the reviewer for suggesting an examination of the distribution of omission responses and for raising the important question of whether these responses form a continuum or distinct groups. In the revised manuscript, we no longer use the preliminary ORN/non-ORN classification. Instead, our analysis directly identifies PEONs based on their probability-dependent omission responses, as described in the Methods and Results (particularly Figure 2A).

The ORN classification was initially based on whether a neuron exhibited a significant omission response in any probability condition. However, as we noted before, this approach introduced statistical confounds related to selection bias and circularity and failed to capture the dynamic, context-dependent nature of predictive coding. Many neurons exhibited omission responses selectively, responding strongly to the omission of one tone (its eventual O_P tone) in high-probability contexts while showing little or no response in other conditions. Because a neuron could be classified as an “ORN” in one condition but as a “non-ORN“ in another, this distinction proved unstable and did not reflect an inherent neuronal property. A simple distribution of omission responses based on this categorization would be misleading, as it would conflate responses from different probability contexts and obscure their true selectivity.

To address the reviewer’s question about whether omission responses form a continuum or distinct groups, we performed a k-means clustering analysis, which identified two clusters: one consisting of neurons with weak or no correlation between omission response and probability, and another with neurons whose omission responses were strongly probability-dependent. The emergence of a high-correlation cluster suggests a distinct population of neurons encoding probability, supporting the validity of the PEON classification. While we do not include a separate figure showing the full distribution of omission responses, the clustering analysis provides a quantitative framework for distinguishing predictive neurons from those that do not encode statistical context.

The relevant section in the Results:

> “Next, we examined whether distinct groups exist based on how strongly each neuron’s omission response correlated with predictability of the O_P tone. To achieve this, we performed k-means clustering on the correlation coefficients and used an unbiased elbow analysis to determine the optimal number of clusters (see details in Methods). The elbow analysis revealed that it’s optimal to cluster the data into two distinct neuronal subpopulations (see the elbow analysis in Supplementary Figure S1): one comprising 771 neurons with little or no modulation of omission responses

by tone probability (mean Spearman's rho = 0.048), and a second comprising 219 neurons exhibiting a robust positive correlation (mean rho = 0.18) (Figure 2A).

Figure 2B illustrates the omission responses of two example neurons across eight different conditions. These range from scenarios with 0% O_P tone (95% O_{NP} and 5% omission) to scenarios with 95% O_P tone (0% O_{NP} and 5% omission). The peri-stimulus time histograms (PSTHs), aligned to the expected onset of the omitted tone, have been baseline-subtracted to isolate the omission response. Neuron 1 (from Cluster 1 in Figure 2A) exhibited only a weak correlation (Spearman's rho = 0.12), while Neuron 2 (from Cluster 2) showed a pronounced increase in omission response as the probability of the O_P tone increased (rho = 0.36)." (page 8 lines 142-157)

The relevant section in the Methods:

"To examine the presence of distinct groups based on the strength of the correlation between each neuron's omission response and predictability of O_P tone, we performed k-means clustering on the correlation coefficients. Thus, neurons encoding predictability exhibited either strong positive or strong negative correlations. To determine the optimal number of clusters, we used the elbow method, which identifies the "knee point" in the plot of within-cluster sum of squares (WCSS) against the number of clusters. The knee point suggests the optimal number of clusters where adding more clusters does not significantly improve the model's fit to the data (see Supplementary figure S1). The k-means clustering analysis was performed using the k-means function in MATLAB with 10 replicates for each cluster number. The WCSS was calculated for each cluster number, and the optimal number of clusters was determined by finding the knee point in the WCSS plot. The clustering results were then visualized using histograms to display the distribution of correlation coefficients within each cluster." (page 36 lines 843-854)

A

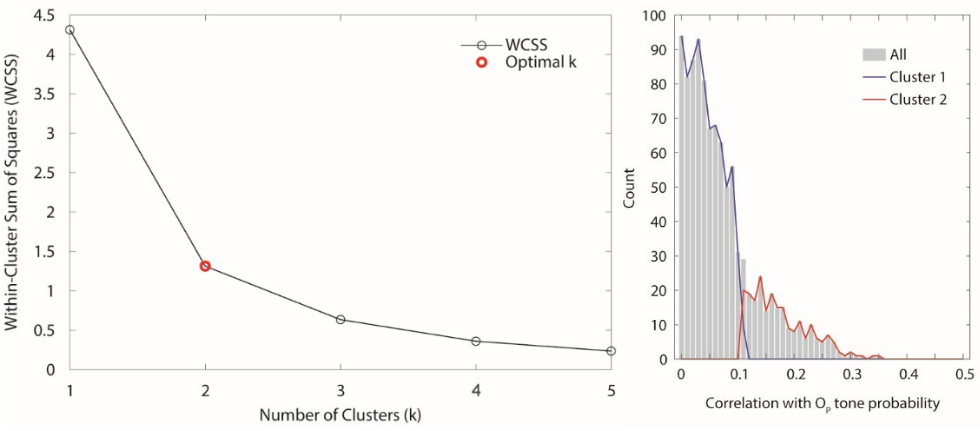

1.4. The authors are focussed in their interpretation on negative prediction error neurons, and center their analysis as well as their interpretation around these hypothetical cells. However, this ignores several alternative interpretations and loopholes in their analysis, most importantly:

(i) The most obvious alternative would be that the omission responses are actually predictions, instead of negative prediction errors. In their final section of the discussion,

the authors comment on the lack of finding predictions encoded in neural responses. While not spelled out very explicitly (L540: "predictions about upcoming stimuli"), I presume that the authors expect predictions to be represented earlier than prediction errors. However, given the slow, build-up time-course of the omission responses observed in the present study, I would say that these either don't fit the prediction errors AND the predictions or both, since differences in the timing between predictions and prediction errors (given the classical inversion in the sensory area, in Fig.7 referred to as I+ neurons) would be expected to be only a few milliseconds. I therefore consider it essential that the authors seriously consider this alternative in the analysis and the discussion. For the analysis, one (but not limited to this) analysis would be to use waveform analysis to check whether the recorded neurons are narrow or wide waveform cells, i.e. whether they are more likely to be inhibitory interneurons or excitatory pyramidal cells.

We thank the reviewer for raising the alternative interpretation that the observed omission responses might represent predictions themselves, rather than negative prediction errors, and for highlighting the important issue of the response time course. The reviewer correctly presumes that we expect prediction signals to be represented earlier than prediction error signals, and, in fact, to exist before the potential onset of the expected stimulus (or its omission). We agree that, in simplified predictive coding models, including the simplified model presented in this study (which focuses on probability encoding and response asymmetry rather than precise temporal dynamics), prediction signals are typically expected to precede prediction error signals by a very short time interval. However, we still believe the PEON activity patterns, considering both the timing of the responses and their occurrence relative to stimulus presentation, strongly support their interpretation as negative prediction error signals.

First, the timing of the response: a signal representing the prediction of an upcoming tone should, by definition, be present by the time the tone is expected to occur. In contrast, a negative prediction error signal arises after the expected time of the stimulus, specifically because the expected stimulus did not occur. The PEON omission responses, by definition, occur after the expected tone onset. Secondly, even if we were to consider a hypothetical prediction signal that, for some reason, is delayed, a pure prediction signal would still not explain the pattern of PEON activity across all trial types. On trials where tone is presented, PEONs often exhibit smaller responses than on omission trials, particularly when the presented tone is highly probable (Figure 4A, reflecting stimulus-specific adaptation). If the omission response were simply a delayed prediction signal, we would expect similar activity regardless of whether the tone was presented or omitted. This reflects the mismatch between expectation and reality, not just the expectation itself.

Added this section to the discussion:

> “It is important to consider whether the observed omission responses might themselves be a prediction signal, rather than solely a prediction error. However, several aspects of the PEON activity argue against this interpretation. First, a prediction signal should precede the expected time of the stimulus, while PEON omission responses occur after the expected tone onset. Second, prediction signals should appear consistently across all trials, regardless of whether the stimulus is presented or omitted. Instead, we observe that PEONs exhibit markedly different activity patterns between presentation and omission trials— in some cases showing smaller responses when tones are presented compared to when they are omitted. Thus, the PEON activity pattern is more consistent with a negative prediction-error signal,

> reflecting the mismatch between expectation and reality, rather than just the expectation itself." (page 30 lines 689-698)

Regarding the latency of the omission response raised by the reviewer: We agree this is an important characteristic. The PEON omission responses exhibit a longer latency than expected by the simplified model presented in this study, which was primarily designed to capture the core features of probability encoding and explain response asymmetry, rather than the precise temporal dynamics of neuronal responses. At the circuit level, this increased latency could potentially reflect dis-inhibition within a recurrent circuit. Functionally, a more gradual increase in the negative prediction error signal, rather than an immediate, transient response, might serve to prevent an immediate "surprise" response upon every brief delay of an expected stimulus. This contrasts with the rapid response required for unexpected stimulus presentations (positive prediction errors), which is critical for behavioral adaptation. The negative prediction error, representing the absence of an expected input, inherently involves a temporal component, potentially related to processes such as a hazard function. We have significantly expanded our discussion of these and other potential mechanisms underlying the increased latency.

The added discussion section:

> "A notable feature of the PEON omission responses is their shape; rather than exhibiting a sharp, transient peak as seen in typical tone responses, they often display a more gradual onset and prolonged duration [26,27,34] compared to both typical auditory evoked responses and the idealized, instantaneous prediction-error signals assumed in simplified predictive coding models, including our own. While our model effectively captures the probability encoding and asymmetry of PEON responses, it does not explicitly model the detailed temporal dynamics. Functionally, a more gradual negative prediction-error signal might help prevent drastic responses to slightly delayed expected input—contrasting with the rapid responses needed for unexpected stimuli. Furthermore, the negative prediction-error signal could reflect a prediction not only about the content (i.e., the tone) but also about its timing. In other words, the prediction signal could be gradually ramped up according to a hazard function, modeling how the brain adjusts its anticipation of an event as time passes [53–56], and the negative prediction-error signal could follow a similar pattern. We suspect that this ramp-up response involves a recurrent cortical circuit normally inhibited by sensory input; when the input is omitted and disinhibition occurs, the negative prediction-error signal gradually increases. Future computational modeling that explicitly incorporates recurrent connectivity and temporal integration mechanisms will be crucial for elucidating the precise neural mechanisms underlying this observed latency." (page 29 lines 661-677)

While we appreciate the reviewer's suggestion to use waveform analysis to potentially distinguish between different cell types (e.g., narrow-spiking inhibitory interneurons and broader-spiking excitatory pyramidal neurons), this was not feasible with the current dataset and time limitation and was not the focus of this current study. Reliable waveform analysis requires exceptionally high signal-to-noise ratio and very stable recordings, to accurately measure subtle differences in spike shape. Due to the constraints of our experimental setup, optimized for detecting and characterizing omission responses across a large population of neurons, the signal quality, while sufficient for identifying single units and analyzing firing rates, was not consistently high enough across all recorded neurons to allow for reliable

waveform classification. We acknowledge this limitation and have included a discussion of the importance of cell-type identification in future studies

> "Another important direction is identifying the specific neuronal populations involved in prediction-error signaling. While our study focused on firing rate dynamics, different neuronal subtypes may play distinct roles in encoding positive and negative prediction errors. Techniques such as optogenetic tagging, juxtacellular recording, or genetic labeling could help determine whether PEONs correspond primarily to excitatory pyramidal neurons or specific subtypes of inhibitory interneurons, providing critical insights into the circuit mechanisms underlying predictive processing in the auditory cortex." (page 30 lines 704-710)

(ii) Related to the question above, the authors build a model on the premise of the existence of positive and negative prediction error neurons, however, in the whole analysis (and discussion, as far as I could find) avoid the question of where the positive prediction error neurons are, which in the case of omitted tones should show negative deviations in firing rate. Given the data in Fig. 2D, it seems that the authors disprove their (widespread) existence, in the areas recorded. This question needs to be addressed much more objectively, and of course has major consequences for the model framework (see below).

We thank the reviewer for raising this important question about the presence and role of positive prediction error neurons in our data. We acknowledge that our stance on this issue could have been made clearer. The reviewer notes that, in the case of omitted tones, positive prediction error neurons should, in principle, show negative deviations in firing rate. However, this expectation does not fully account for the specific characteristics of sensory cortex and the nature of omission responses.

The organization of prediction error signaling in sensory cortex differs from systems like the dopaminergic system (e.g., Schultz et al., 1997). In sensory cortex, prediction error encoding operates with fundamentally different constraints and mechanisms. Specifically, we, and others, generally assume that positive prediction-error neurons in sensory cortices have very low or even near-zero baseline firing rates. Therefore, detecting a further decrease below this already low baseline during omissions is statistically challenging and unlikely to yield interpretable results. Instead, positive prediction error neurons are best identified by their responses to unexpected tone presentations, where they should exhibit increased firing rates. We have indeed found clear evidence for such positive prediction error encoding in our data, in the form of stimulus-specific adaptation (SSA) and deviance detection in the tone responses of neurons not classified as PEONs (non-PEONs). These results are presented in Supplementary Figure S7 and detailed in a new discussion section. As shown in this figure, the average response of non-PEONs to both increases as their probability decreases, reflecting positive error signaling (as previously described by Taaseh et. al 2011, Rubin et al. 2016, parras 2017 and many others).

Therefore, while we do not find evidence for positive prediction error neurons decreasing their firing on omission trials, we do find strong evidence for positive prediction error encoding in the tone responses of non-PEONs. This is consistent with the predictive coding framework, which posits both positive and negative prediction error computations, but it clarifies that, in the context of auditory cortex and omission paradigms, positive prediction errors are most clearly revealed by responses to unexpected tone presentations, not by decreases in firing during omissions.

“Our model builds upon the framework proposed by Keller and Mrsic-Flogel, which describes predictive coding as a canonical cortical computation [6]. Their model emphasizes hierarchical processing with distinct neuron populations computing positive and negative prediction errors. Consistent with this framework, our findings provide evidence for both types of prediction error in the rat auditory cortex. While we focused on characterizing negative prediction errors in PEONs, we also observed oddball responses in the broader population of non-PEON neurons (Supplementary Figure S7), with enhanced responses to unexpected tones. Since such responses have been interpreted as positive prediction-error signals in previous studies, we infer that at least some of these neurons encode positive prediction error [16,18,37–40]. ” (page 24 lines 549-557)

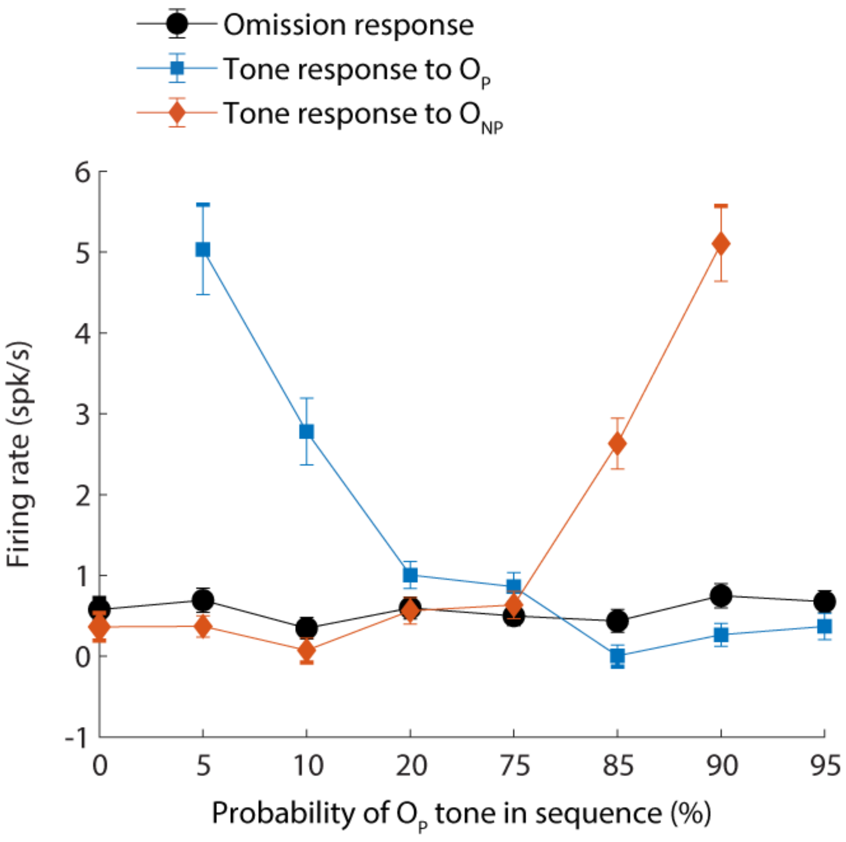

(iii) In both cases I am missing a serious discussion of why the time-course of the omission responses is so slow. In their model the authors use a much faster time-course for the prediction, which seems to be unrelated to any of the data presented here in terms of time course. While the (likely rare) cells in Figure 2B/C show rather rapid onsets of the omission response, there is no decay after the expected sound (similar to the previous study by Lao-Rodriguez et al.) which seems at odds with the classical predictions for prediction errors and predictions.

We appreciate the reviewer’s concern and agree that the slow time-course of omission responses requires further discussion. The absence of decay after the expected sound, as observed in Lao-Rodríguez et al. (2023,2024), suggests that omission responses integrate information over longer timescales rather than reflecting an instantaneous prediction error. While quite a few cells do show rapid onsets, the dominant pattern points to a slower,

integrative process. As noted before, we have expanded our discussion to reflect these considerations as described in our response to comment 1.4.

1.5. The finding of probability encoding would be a very interesting finding, however, needs some clarification. In Fig. 4B, the y-axis is indicated as firing rate in the axis label and the caption, and I assume this related to response to the omission, but this needs to be spelled out clearly. However, if this is the case, then the firing rate effect size does not seem to match, comparing with Fig. 2D (~10-15Hz over the whole period) with the present data (~1-2Hz).

We thank the reviewer for their careful attention to detail regarding Figure 4B (now Figure 2B in the revised manuscript) and for pointing out the discrepancy in firing rates compared to the original Figure 2D. We confirm that the y-axis in Figure 2B represents the baseline-subtracted firing rate in response to the omission, calculated over a 5-120ms window after the expected tone onset. We have revised the figure legend to make this explicit.
Regarding the firing rate differences, the reviewer is correct in noting a discrepancy between the firing rates shown in the original Figure 2D and the current Figure 2B. The original Figure 2D presented grand average PSTHs for neurons classified as ORNs and non-ORNs. We acknowledge that this initial ORN/non-ORN classification, and the associated figure, contained an error. While the qualitative pattern of responses in that figure was correct, the quantitative values were incorrect due to an error in the summation. That said, the figure was removed from the revised manuscript primarily for a different reason—our refined analysis framework no longer relies on this specific ORN/non-ORN classification, as outlined in our response to comment 1.

1.6. The finding that omission responses are specifically related to particular stimuli in individual neurons is interesting, but an essential piece of information is missing: how are the selectivities of the cells to A/B, related to the omission responses, i.e. with the subquestions:
(i) if one relates the tuning curve of each cell to the selectivity of A/B, do they match? (I guess they do, but on the other hand, the way the stimulus was chosen, as classically done, centered around the mean preference of the cells, means that the lack of specificity to A/B/broad response is partly by design)
(ii) If one correlates the response to A and B with the Omission response to sequence with dominantly A or B, per cell, does one find positive or negative, or no relation? I understand that Fig.6C (left) suggests no relation, but this could be a consequence of the criteria for the assessment of selective/non-selective, and a correlation might be a more direct way of showing this.

(i) Tuning Curve Match:
The reviewer is correct that the selection of Tones A and B was based on the overall responsiveness of the recorded population, as is common practice in auditory neurophysiology experiments. We first used the Neuropixels electrode to map the frequency response area (FRA) across the recorded neurons. From this initial mapping, we selected two distinct tones, one octave apart, that consistently elicited robust responses from a majority of the recorded neurons. This procedure is detailed in the Methods section. While we did not retain a full FRA for each individual neuron after this initial mapping (due to the difficulty in reliably tracking individual neurons across different stimulus sets), we do have the responses to the selected tones A and B for each neuron. Our initial mapping ensured that both tones were within the generally responsive range of the recorded population. Therefore, the broad tone responses observed in many PEONs are, to some extent, by design.

> “To assess the frequency-tuning properties of all recorded neurons, we measured the frequency response area (FRA) at each recording site. The auditory stimuli consisted of pure tone bursts with a 5-ms rise/fall time and a 20-ms steady-state duration. The tones covered a wide range of frequencies, spanning 18 distinct values from 1.6 to 64 kHz, and were presented at seven different sound pressure levels, ranging from 20 to 80 dB SPL in 10-dB steps. In total, 126 unique tone bursts were used to characterize the FRA. The stimuli were delivered in a pseudorandom sequence with an inter-tone interval of 600 ms, and each tone was repeated 20 times. FRA maps were generated for each neuron based on their responses to the presented tones. After examining the FRA maps, we selected two tones that were one octave apart and elicited robust responses from most recorded neurons. These two tones were then used as the stimuli for the subsequent experiments investigating the neural responses to tone sequences and omissions using an intensity well above the threshold of a typical neuron.” (page 34 lines 791-802)

(ii) Correlation between Tone Responses and Omission Responses:
We thank the reviewer for this suggestion and for pointing out the potential limitations of using unly the categorical classifications presented in the original Figure 6C (left) (now Figure 4C (left) in the revised manuscript). We agree that an additional direct correlation analysis may be a useful quantitative way to assess the relationship between tone selectivity and omission selectivity.

As suggested, we performed a correlation analysis at the single-neuron level to test whether a neuron’s preference for the presentation of a tone predicts its preference for the omission of that tone. The rationale for this analysis, and the detailed methodology, are now included in the revised Results and Methods sections. Briefly, we computed a Tone Selectivity Index (TSI) and an Omission Selectivity Index (OSI) for each PEON. The TSI quantifies the neuron’s relative response to Tone A versus Tone B presentations, averaged across conditions where each tone was presented. The OSI quantifies the neuron's relative response to omissions in sequences where Tone A was the O_P tone versus sequences where Tone B was the O_P tone. We then calculated the Pearson correlation coefficient between TSI and OSI across the population of PEONs.

The results of this analysis, now included in the Results section, revealed no significant correlation between TSI and OSI (Spearman’s rho = –0.03, p = 0.78, n=123 Neurons) . This finding indicates that a neuron's preference for the presentation of a specific tone does not predict its preference for the omission of that tone, suggesting that tone-evoked selectivity and omission selectivity are largely independent properties within the PEON population. This supports the interpretation that these two types of selectivity arise from distinct neural mechanisms, rather than a shared, bottom-up encoding process. This null result is presented in addition to, and complements, the categorical analyses shown in Figure 4C.

In the Results section:

> “To ensure that the selectivity observed in omission responses is not merely due to residual effects from the previous trial’s tone responses—such as lingering activity or altered baseline activity—we verified that omission selectivity is independent of tone selectivity. To do this, we measured the omission selectivity index (OSI) and the tone selectivity index (TSI) for each PEON (see details in Methods). The results showed no significant correlation between OSI and TSI across the PEON population (Spearman’s rho = –0.03, p = 0.78, n=123 Neurons). This lack of correlation indicates

that a PEON's preference for a specific tone (e.g., Tone A over Tone B) does not predict its preference for the omission of that tone. Therefore, omission selectivity was not a residual effect from the previous trials. " (page 13 lines 275-283)

In the Methods section:

"To assess the relationship between tone-evoked selectivity and omission selectivity, we computed selectivity indexes - TSI and OSI for each neuron. TSI was calculated as:

$$TSI = \frac{T_{O_P} - T_{O_{NP}}}{T_{O_P} + T_{O_{NP}}}$$

T_{O_P} and $T_{O_{NP}}$ represent the neuron's mean firing rate in response to the O_P and O_{NP} tones, respectively, averaged across the seven probability conditions in which each tone was presented. OSI was calculated as:

$$OSI = \frac{O_{O_P} - O_{O_{NP}}}{O_{O_P} + O_{O_{NP}}}$$

O_{O_P} and $O_{O_{NP}}$ denote the neuron's mean firing rate in response to omissions occurring in the 4 sequences where the O_P and O_{NP} tones were standard, respectively.
To test whether a neuron's tone selectivity predicted its omission selectivity, we computed Spearman's correlation between TSI and OSI." (page 39 lines 922-932)

1.7. *Regarding the proposed model. While I find it commendable that the authors compare their model to alternative models (and the different probabilities are the most useful parameter here for distinguishing), I think the proposed model is only very loosely based on data provided in the present publication and receives too much weight, given a low level of evidence for it. Specifically, I think the structure of the model is more or less made up to fit a part of the data, rather than in detail based on the data, in particular*
(i) the lack of evidence for positive prediction error neurons (which according to the model should be present in equal proportions, I suppose)
(ii) the lack of evidence for different cell types, which perform different functions in the model
(iii) the use of unrealistic temporal waveforms for the prediction (at least in relation to the data shown), without a measurement of these waveforms in other areas.
(iv) no direct evidence for any lateral connections based on the data, e.g. by using correlation analysis between neighboring locations in cortex etc.
(v) If PE- neurons in stream 2 receive inhibitory input from Sensory Signal A with a greater weight than the lateral connections from the neighboring streams, why can they still show a positive response to the Stimulus A (see Fig. 2D)? Or are you assuming that the sum of the input from the neighboring columns exceeds the local positive input? This would be a very interesting outcome, but seems unlikely given the typical connectivity patterns of input into cortex, and thus seems quite a stretch to propose as a basis for the model (given the responses in 2D).
Therefore, while the outcomes of the model are in some agreement with the data, I think the model is more likely to mislead readers into putting trust into a not-well-supported model, rather than interpreting the data as is. Therefore, I would strongly suggest to reduce the prominence of the model in the manuscript and relegate it to a supplement or publish it in a more theoretical journal.

We acknowledge the reviewer's concerns about the model's level of detail and direct empirical support, and we have revised the manuscript to emphasize its role as a *hypothesis*

that provides a *potential* circuit-level explanation for the observed asymmetry, rather than a fully validated model. The purpose of the model is indeed to explain the data, and it is based on the framework proposed by Keller and Mrsic-Flogel, which is the only framework that describes how negative prediction errors are presented and integrated in neural circuits. One key element missing in their model is whether and how positive and negative prediction errors interact across sensory streams. This aspect is crucial to explain our data, which is why we propose multiple ways to integrate these streams and evaluate them with our data. We believe that our model not only provides the first theoretical circuit model connecting the prediction of different contents with enhanced prediction encoding but also offers a testable hypothesis for further experimentation.

In the revised Results section, where the model is first introduced, we highlight its inspiration to clarify that it is not entirely new or made up:

> "To further understand how PEONs, which we presume to be negative prediction-error neurons, can display this observed asymmetry in top-down and bottom-up processing, we developed a neural circuit model consisting of multiple streams within a "predictive-coding module" (Figure 5A). This model was based on the framework proposed by Keller and Mrsic-Flogel, which describes a circuit module that integrates negative prediction error computation [6]." (page 14 lines 308-312)

Below, we address the reviewer's concerns point by point:

(i) Lack of evidence for positive prediction error neurons:
As addressed in our response to comment 1.4(ii), while our primary experimental focus was on negative prediction error, we do present evidence of positive prediction error encoding in the tone responses of non-PEON neurons (Supplementary Figure S7). As shown in this figure, the average response of non-PEONs to tones increases as their probability decreases, reflecting positive error signaling. This is consistent with previous work demonstrating that neurons in the auditory cortex exhibit stimulus-specific adaptation and deviance detection, which are considered signatures of positive prediction error encoding.
(ii) Lack of evidence for different cell types:
As already discussed in our response to comment 1.4(i), while we did not directly identify distinct cell types corresponding to each model component, the model's functional units represent hypothesized roles within a predictive coding circuit, consistent with broader theoretical frameworks. We have acknowledged this limitation and discussed potential future directions for cell-type specific investigations in the revised Discussion section.

(iii) Unrealistic temporal waveforms for the prediction:
As already addressed in our responses to comments 1.4(i) and 1.4(iii), the model's temporal dynamics were simplified to focus on probability encoding and response asymmetry. We acknowledge that future, more biophysically realistic models should address the observed response latencies. This is now explicitly discussed in the revised manuscript where we explain the limitations of our simplified temporal dynamics and potential functional reasons for the gradual onset and prolonged duration of PEON omission responses.

(iv) No direct evidence for any lateral connections:
While we did not directly measure lateral connection strengths in this study, the proposed lateral connections are consistent with known anatomical and physiological properties of auditory cortex, serve to explain the observed asymmetry, and, importantly, generate testable

hypotheses for future experimental investigations aimed at elucidating the precise circuitry underlying negative prediction error computations.
As we discuss in the revised Discussion section:

> "Biological evidence strongly supports the existence of excitatory lateral connections between adjacent frequency channels in A1. These connections play a crucial role in spectral integration and the processing of complex auditory stimuli [42–44]. Anatomical studies have revealed that pyramidal neurons in A1 exhibit long-range horizontal collaterals that form connections across different frequency representations, often aligning with isofrequency bands [45,46]. Physiological investigations have demonstrated that these intracortical pathways contribute significantly to the breadth of subthreshold frequency receptive fields, which can span up to five octaves [47,48]. The strength and probability of these connections are distance-dependent, with stronger and more probable connections between neurons tuned to close frequencies, weakening as the frequency distance increases [49,50]. This organized network of lateral connections facilitates the integration of spectral information across different frequency channels, supporting complex auditory processing and cortical plasticity in A1" (page 25 line 580 - page 26 line 591)

(v) PE- neurons in stream 2 receive inhibitory input...:
Yes, the positive response of PE- neurons to Tone A, despite local inhibition, is a result of the *combined* excitatory input from lateral connections driven by *positive* prediction errors in neighboring streams responding to the unexpected Tone B. In our simplified model, which includes only two neighboring streams, the lateral excitatory weights (the strongest being 1.2; see Figure 5E, right panel) must exceed the inhibitory weight (0.5; see Table S1). With more lateral neighbor neurons, these excitatory weights can be reduced. Moreover, we propose that these lateral connections could be activity-dependent (e.g., via spike-timing-dependent plasticity) and further refined through self-organization. However, this lies beyond the scope of our current study, and we address it in the revision.

> "Note that this asymmetry requires that the weights of the lateral excitatory connections from nearby neurons (as shown in Figure 5E) exceed the inhibitory weights within the module (see Table S1). This setup ensures that sensory inputs from the neighboring streams can overcome inhibition to elicit a response on PEONs. When the number of lateral neighbor neurons increases (there are only two in our model), reduced excitatory weights can remain effective. Furthermore, we believe these lateral connections could be activity-dependent (e.g., via spike-timing-dependent plasticity) and further refined through self-organization. However, this lies beyond the scope of our current study." (page 18 lines 400-407)

1.8. The control performed in supplementary figure 1 seems very insufficient to me, although generally of high relevance. Qualitatively one can already see that a similar behavior starts to emerge, and it is therefore hard for me to assess whether this is just a matter of the parameter settings chosen here. Therefore the choice of parameters of the adaptation needs to made much more explicit and related to the amount of adaptation typically observed. After all, the responses to A and B across different Prob A conditions are essentially deviant/oddball responses, which are expected to vary with Prob A/B. Previous research has shown that this can already be explained largely by local adaptation in response (e.g. Tasseh et al. 2011). Previous research has shown the adaptation is sometimes not top-down modulated (Lesicko et al. 2022, in the IC), and it is therefore an option that needs to be seriously explored.

We thank the reviewer for the suggestion and performed additional simulations with various levels of adaptation, controlled by an adaptation factor *a* and a baseline activity level *b*. The results are shown in the revised Supplementary Figure S6 (attached below). Even though the *S_strength* changes drastically across different combinations of *a* (0.01, 0.05, 0.1, and 0.5) and *b* (0.1, 0.3 and 0.5) (panel B), the response curves do not change much (panel C). Still, the model with "Lateral PE+ to PE–" remains the best match to the experimental results.

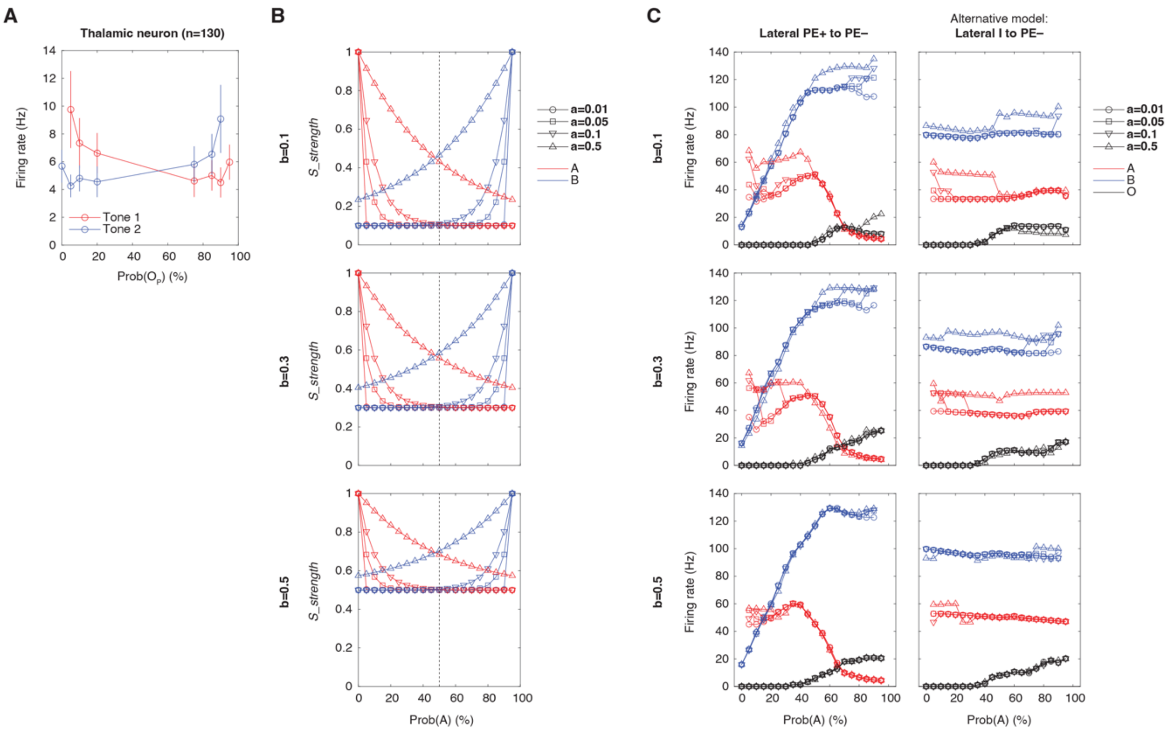

We realized that the detailed methods for modeling sensory adaptation were missing in the previous version, so we have added them in the revision:

> "To model sensory adaptation, we adjusted the input I_{stim} by using an adaptation factor a, which is greater than zero, and a base firing factor b, which is between 0 and 1. The input strength for the A and B streams under different values of Prob(A) was adjusted as follows:
>
> $$S_{strength_A} = (1-b) * e^{Prob(A)/a} + b$$
> $$S_{strength_B} = (1-b) * e^{(1-Prob(A))/a} + b$$
>
> Examples of $S\_strength_A$ versus Prob(A) are shown in Figure S6, with 12 combinations of a (0.01, 0.05, 0.1, 0.5) and b (0.1, 0.3, 0.5)." (page 41 lines 962-968)

Minor Points:
1.9 - L235: "intriguing asymmetry between top-down and bottom-up processing within the PEON population." As the present study did not show any top-down/bottom up differences on a physiological level, this is speculation at this point and should be removed or rephrased.
We thank the reviewer for pointing out the speculative nature of the original phrasing. We have revised the sentence to remove the explicit reference to top-down and bottom-up processing and instead focus on the observed *functional* asymmetry. The sentence now reads:

> "In summary, these characteristic responses—observed in a considerable subpopulation of individual PEONs, which are selective for omissions but broadly

> responsive to tones—mirror the population responses and indicate an intriguing functional asymmetry. This raises the question of how computational principles and circuitry give rise to this phenomenon." (page 14 lines 301-305)

1.10 - Why label the same stimuli in the model A/B and the experimental data O_P and O_NP, after all they are then treated as analogues in Fig. 9.

Tone O_P and O_{NP} are defined functionally. Therefore, we believe it's more appropriate to describe the model first with tone A and B, which are simply different tones for different streams.

1.11 - Fig. 7: clearly label the streams and the predictive coding module

We thank the reviewer for this suggestion. We have revised Figure 5 (previously Figure 7) to clearly label the components.

1.12 - Fig. 5: Add a second panel normalized to the auditory neurons, to better compare the fraction of neurons that show a PEON type across layers.

We thank the reviewer for this suggestion. In our revised manuscript, we have substantially changed our analysis framework by eliminating the preliminary classification of 'auditory neurons' to avoid potential selection bias. Instead, we now identify PEONs directly from the entire recorded population using our split-half approach.
In Figure 2E (previously Figure 5), we now show the laminar distribution of PEONs as a percentage of the total recorded neurons within each layer, allowing for a direct comparison of PEON prevalence across layers. This approach provides a more accurate representation of the laminar distribution without introducing potential biases from pre-selection criteria. The figure clearly illustrates the higher proportion of PEONs in the supragranular (16.2%) and granular (13.4%) layers compared to the infragranular layers (9.8%). as stated in the Results section.

1.13 - Fig. 3 Dynamics of Omission response build-up: The x-axis is labelled trial, which I interpret as omission in side the sequence of 1000 stimuli (including omissions). Since one would expect the buildup to be dependent on the sequence regularity/length, rather than k-th omission, wouldn't it make more sense to show the buildup as a function of sequence position (when the omission occurred)? This could reduce the variability, while I agree that the relation is quite clear.

We thank the reviewer for this suggestion. We have updated Figure 3A to plot the omission response magnitude as a function of sequence position (binned omission trial number), as suggested. The x-axis label and figure legend have been updated accordingly
Methods:

> "For each PEON and each repetition, omission responses were extracted from the four conditions in which the O_P tone was presented as the standard (i.e., 75%, 85%, 90%, and 95% O_P tone). The omission responses were binned based on their sequence position, and for each bin the mean firing rate and standard error were computed, yielding a two-dimensional response matrix that captures the temporal evolution of omission responses.

The mean omission response was then modeled with a logistic growth function using MATLAB's nlinfit function. The model was defined as: FR(n) = a / [1 + exp(–b × (n – c))], where n represents the bin center corresponding to the sequence position, and a, b, and c are free parameters estimated from the data. The goodness-of-fit was assessed by calculating the R-squared value based on the residual and total sums of squares." (page 38 lines 895-905)

Results:

"To confirm that PEONs encode negative prediction errors, it is essential to show that their responses to omissions increase over time as predictions are gradually formed, reflecting a growing discrepancy between the predicted and actual outcomes. We analyzed $PEON_{ODD}$ from EVEN trials and focused on omissions of the O_P tone in sequences where it was the standard (75%, 85%, 90%, and 95%), and therefore expected to evoke the largest omission responses. We grouped these responses by their sequence position and computed the mean firing rate (± standard error) for each bin. This grouping tests whether the omission response increases as more tones precede the omission, which is the expected pattern if these neurons are indeed signaling a negative prediction error that strengthens with increasing prediction.

Our analysis revealed a notable pattern: the magnitude of the PEONs response to these omissions progressively increased throughout the sequence, achieving a steady state or plateau after approximately 400 standards were presented (Figure 3A). The dynamics of the trial-by-trial omission response were fitted by a logistic growth curve, expressed as:

$$FR(n) = \frac{4.95}{1 + e^{-0.012*(n-223.28)}}$$

where $FR(n)$ represents the firing rate as a function of n, and n is the bin center corresponding to the sequence position of the omissions (i.e., the number of tones presented preceding each omission). The logistic function depicts a saturating increase in response magnitude. Here, an R^2 value of 0.51 indicates the model's moderate success in capturing the variability in responses." (page 11 lines 218-237)

1.14 - In its current form the (potentially very interesting) treatment of the free-energy principle derivation, stays hidden in the supplement. I would suggest to extend the current section and add a corresponding figure that visualizes the relations, or remove the section altogether from the results.

We thank the reviewer for highlighting the importance of the free-energy principle derivation. We agree that this theoretical framework provides valuable context for our findings. Following the reviewer's suggestion, we have removed the brief section from the Results and expanded it into a dedicated subsection in the Discussion titled "Lateral Prediction Suppression and the Free Energy Principle." This expanded discussion more thoroughly integrates the theoretical framework with our experimental observations and model, while maintaining the paper's focus on our empirical findings. The detailed mathematical derivations remain in the Supplementary Materials for readers interested in the complete formalism, but the Discussion now provides a more substantive treatment of how our observed lateral prediction suppression naturally emerges from free energy principles. This reorganization improves the paper's flow while giving appropriate prominence to this important theoretical connection.

Discussion:

"Building upon these anatomical and functional observations of lateral connectivity, we can consider how our proposed circuit model might be interpreted through theoretical principles. The free energy principle [1,32] potentially offers an elegant explanatory framework for understanding why these lateral connections may have evolved. Could the lateral prediction suppression we observed be more than just a convenient circuit mechanism? When viewed through the lens of free energy minimization, these connections might represent an optimal solution for efficient predictive processing. In the Supplementary Information, we provide a mathematical treatment that suggests when a simplified form of the free energy principle is applied to a two-tone paradigm similar to our experimental design, lateral interactions between prediction errors across different streams emerge naturally from the formalism.

In our mathematical exploration, we considered a simplified generative model where auditory tones at different frequencies are represented as hidden variables with linear relationships to sensory inputs. By deriving the update rules for prediction signals under free energy minimization, we found that the resulting equations comprise three key components: updates based on prediction errors within the same frequency stream, lateral interactions of prediction errors across different frequency streams, and a temporal decay term. Notably, the lateral interaction term emerges from the correlation structure of sensory inputs across different frequencies—a statistical regularity well-documented in natural auditory environments [52]. These theoretical derivations align remarkably well with our proposed circuit model, where lateral connections between PE+ and PE- neurons across different frequency streams play a crucial role in shaping prediction signals.

This theoretical perspective raises intriguing possibilities about why the brain might implement the asymmetric receptive fields we observed. The efficient coding strategy—where unexpected stimuli in one frequency channel not only signal prediction errors within that channel but also laterally modulate predictions in neighboring channels—could represent an evolutionarily advantageous solution for minimizing computational costs while maximizing predictive accuracy. Such a theoretical framing might also explain the directionality of lateral connections we proposed, flowing primarily from positive to negative prediction error neurons rather than bidirectionally. While experimental validation of these theoretical predictions remains a challenge for future work, the mathematical alignment between our empirical findings and free energy principles suggests that lateral prediction suppression could be a fundamental computational motif in predictive processing, potentially extending beyond the auditory system to other sensory modalities" (page 27 line 612 - page 28 line 646)

1.15 - Discussion: the difference in the fraction of neurons found in this present study compared to Lao-Rodriguez et al. 2023, is related to the use of NeuroPixel electrodes and the use of different probabilities. As the quantitative difference is rather substantial, could the authors elaborate more on these potentially explanatory differences, as I found neither of them to be convincing.

We thank the reviewer for raising the question of differences in the proportion of omission-responsive neurons compared to Lao-Rodríguez et al. (2023). We have removed the direct numerical comparison from the revised manuscript, as we believe it is not appropriate due to significant differences in methodology and neuron classification criteria. A direct comparison of percentages is misleading for several reasons.

The most crucial difference lies in how omission neurons are defined. Lao-Rodríguez et al. classified neurons as "omission-sensitive" based on a significant response in one experimental condition. Our study, in contrast, now focuses on PEONs, which are identified based on a correlation between omission response magnitude and tone probability across multiple conditions, as well as a significant response in the high probability O_P conditions. This is a qualitatively different criterion, specifically designed to identify neurons that encode negative prediction error in a probability-dependent manner.
Furthermore, while both studies used extracellular recordings, our use of Neuropixels probes resulted in simultaneous recording from a large population of neurons across cortical layers including neurons that do not respond to auditory stimuli at all. Furthermore, our use of Neuropixels probes enabled simultaneous recording from a large, unbiased sample of neurons across cortical layers. This contrasts with approaches that rely on sequential recordings from individual neurons, or from smaller arrays. While our initial attempt to pre-select 'auditory neurons' was intended to focus on task-relevant cells, we ultimately removed this pre-selection to avoid any potential bias and to ensure that our PEON classification was based solely on the omission-response properties. These differences in approach, combined with the differences in experimental paradigm and neuron classification, make direct numerical comparisons of neuron percentages inappropriate.

Reviewer #2

Yaron et al., used extracellular recordings to study omission responses in the auditory cortex of anesthetized rats, aiming to reveal which information is encoded in these responses and proposed a circuit model to replicate the experimental findings. Based on their results they formulate an interesting hypothesis for an extension of the classical predictive processing circuit in cortex. While the experiments are well designed and address the question of probability encoding by auditory cortex neurons, some of the claims in the manuscript are not adequately supported by appropriate analysis of the data. Assuming the authors can address our concerns, we would recommend publication.
This review was written in collaboration with a postdoc in the lab.

We thank Reviewer 2 and their colleague for their careful reading of our manuscript, their positive assessment of the experimental design and the interest of the hypothesis, and their constructive criticism.

2.1. Figure 2D - The authors appear to be selecting ORN based on omission responses and then quantifying the strength of these responses on the same data. This is circular analysis (or double dipping) and is prone to regression to the mean type artefacts. Equivalent would be grouping people in two groups by height and then concluding that one group is significantly taller than the other on average. The authors select neurons with significant responses to omission (line 135) and then conclude that they have a significantly stronger response to omissions (line 146). This is circular of course. One way to deal with this problem, is to split the data in half (by trials) and select and analyze on half of the trials each. E.g. ORN can be selected based on even trials, and then odd trials can be used to quantify their omission responses. This avoids the most prevalent regression to the mean artefacts.
2.2. Figure 4B - There is a regression to the mean issue here as well. PEONs are defined as ORNs that show a significant correlation between omission responses and the percentage of Op tone, but then a linear mixed-effects model is applied only to the neurons that had been selected to have significant correlations. The model should either be applied to all ORNs, or selection should be conducted on half of the data (trials), with statistical testing performed on the other half of the data (trials).

We thank the reviewer for raising this critical issue regarding potential selection bias in our analysis, particularly in the classification of ORNs. As noted, selecting neurons based on their omission responses and then quantifying those same responses on the same dataset introduces circularity and can lead to regression-to-the-mean artifacts. Similarly, in Figure 4B, PEONs were defined based on a significant correlation between omission responses and O_P tone probability and then analyzed using a linear mixed-effects model applied only to this pre-selected subset, which could inflate the observed effect size.

To address these concerns, we have fundamentally revised our analysis by eliminating the ORN/non-ORN classification, which also led to the removal of Figure 2D. Instead, we now identify PEONs directly based on their probability-dependent omission responses. Furthermore, we implemented a split-half approach to ensure that neuron selection and characterization are performed on independent data. Trials were divided into two independent subsets—odd and even trials—where one subset was used to classify PEONs and the other was used to quantify omission responses. This prevents circularity by ensuring that selection

and characterization are independent. An additional analysis using all trials was conducted to confirm the robustness of our conclusions.

These changes are reflected in the updated Figure 2D, which now replaces the previous Figure 4B, and its description in the Results section. The Methods section has also been revised to detail the split-half procedure, and supplementary figures provide further validation of these findings. While these refinements altered the specific number of neurons classified as PEONs and some statistical values, the overall conclusions of the study remain unchanged. Below, we provide a detailed account of the specific changes made:
In the Methods section, we added a section on the split-half analysis:

> "To mitigate potential biases such as double dipping—where the same data are used both to select and evaluate effects—and to ensure the robustness of our probability encoding analysis, we employed a split-half approach. For each probability condition, the 50 omission trials were divided into two equal halves—an odd-indexed "training set" and an even-indexed "testing set," with each set providing 25 omission trials per condition. Using only the training set, we computed Spearman's rank correlation between each neuron's omission response and the probability condition. neurons with a significant correlation ($p < 0.05$) were provisionally labeled as "correlated," and the O_P tone for each neuron was defined based on the direction of the correlation in the test data. From this subset of correlated neurons, we then pooled the training-set trials in the conditions where the O_P was standard (75–95%) and applied a one-sample Wilcoxon sign-rank test ($p < 0.05$) to determine whether their omission response was significantly above zero. Because the omission response is computed as the firing rate minus a local baseline, testing against zero effectively assesses whether post-omission activity exceeds the neuron's immediate pre-omission baseline. Only neurons that exhibited both a significant correlation and a robust omission response were designated as PEONs in the training set. All subsequent population plots—such as boxplots or group correlations—were generated solely from the testing set, ensuring that any observed trends were unbiased by the training-set selection process. Complementary analyses were also carried out using even-indexed trials for training (with odd-indexed trials for testing) as well as a non-split method utilizing all trials; these additional analyses, presented in the Supplementary Materials, confirm that our main conclusions do not depend on the specific trial split." (page 36 line 857 - page 37 line 877)

This change resulted in an update to Figure 2. The new split analysis is now reflected on the figure and corresponding text in the results section:

> "To further examine these PEONs without introducing double-dipping biases, we conducted a split-data analysis by dividing trials into two independent subsets (odd and even trials, denoted as ODD and EVEN trials) to ensure the robustness of our analyses. Using the ODD trials, we identified 123 PEONs according to two criteria: (1) a significant correlation ($p < 0.05$) between their omission responses and the probability of the corresponding O_P tone, as verified by Spearman's correlation test; and (2) a significant omission response ($p < 0.05$, Wilcoxon sign-rank test) to this OP tone in the four conditions where it was presented as standard (75%, 85%, 90%, and 95%), when the O_P tone is most predictable and omission responses should be greatest (see details in Methods). We refer to these PEONs as $PEON_{ODD}$. To confirm the reliability of our selection criteria and ensure that the identified neurons were not specific to one trial subset, we applied the same classification procedure to the EVEN trials, identifying a separate group of PEONs referred to as $PEON_{EVEN}$. For additional

comparison, maximizing statistical power and evaluating probability encoding without trial-based data splitting, we identified $PEON_{ALL}$, a group classified using all available trials." (page 8 line 159 - page 9 line 172)

Furthermore, the split-half analysis was incorporated into Figures 3, 4, supplementary figures 2-5, and their corresponding text, to ensure consistency and rigor across all analyses involving PEONs.

Some of the conclusions throughout the paper are overstated and should be refined for clarity and precision.
2.3. In l.90 " (...) and offer a comprehensive account of how these errors are computed and integrated. " "comprehensive" is probably a bit strong - the existing model is extended with one additional connection. The circuit diagram is likely far from complete.

We thank the reviewer for pointing out the overly strong phrasing. We agree that "comprehensive" was an overstatement. We have revised the sentence to read:

> "Our findings provide empirical evidence for the existence of negative prediction error neurons in the auditory cortex and propose a circuit-level hypothesis for how these errors might be computed and integrated within cortical microcircuits." (page 4 line 88 - page 5 line 91)

2.4. l. 482 "Our study provides empirical evidence for these mechanisms in the auditory cortex, extending previous findings on negative prediction error neurons in visual cortex [7,9] to the auditory domain." - This again is a bit strong, prior work has already identified negative prediction error responses in auditory cortex e.g. https://pubmed.ncbi.nlm.nih.gov/37699716/ or https://elifesciences.org/reviewed-preprints/95398.

We thank the reviewer for pointing out that the original phrasing of this sentence was too strong and for bringing to our attention the relevant prior publications. We have removed the sentence from this subsection and added further discussion of the shoulders we stand on in the prior section and in introduction:

> "Omission paradigms are a powerful tool for isolating negative prediction-error signals, as they eliminate the confounding influence of sensory input present in positive prediction error paradigms (e.g., oddball sequences). In human studies using EEG and MEG, mismatch negativity (MMN) responses to unexpected omissions in tone sequences have been reported, which are interpreted as evidence for predictive coding mechanisms [17,20,22–24,32]. More recently, single-neuron recordings in animals have begun to reveal the neural mechanisms underlying these auditory prediction errors. Studies have reported omission-responsive neurons in the auditory cortex of both awake and anesthetized rodents. One study found that 35.6% of neurons in awake animals and 19.5% of neurons in anesthetized rats and mice exhibited significant responses to the omission of expected tones [33,34] Another study, using more complex stimuli involving the omission of expected gaps within auditory sequences, also demonstrated robust omission responses in unanesthetized rats.[25]. Alongside these auditory findings, multiple studies in mouse visual cortex have documented sensorimotor prediction-error signals, specifically negative prediction errors, in response to unexpected reductions in expected sensory input [7,35,36]. These studies demonstrate that neurons in V1 exhibit negative mismatch responses when expected visual flow is weakened or omitted during locomotion. A

molecularly distinct population of neurons in V1 has been identified that selectively responds to unexpected omissions of predicted visual stimuli[9] Extending this sensorimotor framework to the auditory system, a recent study showed that neurons in the auditory cortex responded to unexpected reductions in movement-contingent sound within a virtual reality environment, signaling a negative prediction error [28]. Building upon this foundation, our study's conceptual advance stems from our paradigm design that systematically varies tone probabilities while maintaining consistent omission rates, allowing us to characterize negative prediction error as a continuous, probability-encoding mechanism rather than simply a binary deviance detector. This approach also reveals the dynamic nature of these prediction signals, which develop gradually through repeated exposure to statistical patterns in the environment. The subsequent sections will explore how these findings, along with our observations of asymmetry in predictive processing, led us to develop a circuit-level model that offers a new perspective on information flow in predictive coding networks. (page 23 line 518 - page 24 line 546)

Caveats in analysis:
2.5. Specifying percentages of omission-responsive neurons, such as reporting 48%, is misleading because this number is derived after first selecting auditory-responsive and omission-responsive neurons, and then identifying omission-responsive cells from within this subset. It would be more accurate to compare these percentages to the entire recorded population. For example, in line 469, the statement 'our study identified a higher percentage of omission-responsive neurons (48% in anesthetized rats)' is misleading. A correct comparison would involve the total number of recorded cells: 411 out of 1,489, yielding 27%, which is more consistent with other reports. 6. Figure 5 - This would be more informative if presented as a percentage of all recorded cells. Additionally, are omission-responsive neurons excluded from the auditory-responsive group, or are some neurons counted in both groups? Similarly, for PEONs and ORNs, are PEONs excluded from the total count of ORNs presented?
2.6. Figure 5 - This would be more informative if presented as a percentage of all recorded cells. Additionally, are omission-responsive neurons excluded from the auditory-responsive group, or are some neurons counted in both groups? Similarly, for PEONs and ORNs, are PEONs excluded from the total count of ORNs presented?

We thank the reviewer for highlighting these points regarding neuron classification, percentage reporting, and the presentation of data in Figure 5 (now Figure 2E in the revised manuscript). We have made substantial revisions to address these concerns.

The reviewer correctly points out that reporting percentages based on pre-selected subsets of neurons can be misleading. In the original manuscript, we included preliminary classifications of 'auditory neurons' and ORNs. We now recognize that these classifications were unnecessary and potentially confusing. In the revised manuscript, we have eliminated all preliminary classifications. We now identify PEONs directly from the entire recorded population, using a split-half analysis to avoid circularity. Therefore, the percentages of PEONs reported in the revised manuscript (e.g., in the Results section: 'Out of a total of 990 recorded neurons, we identified 123 (13%) $PEON_{ODD}$') are calculated relative to the total number of recorded neurons, not to any pre-selected subset.

Furthermore, we now avoid direct numerical comparisons between the prevalence of PEONs in our study and the percentage of omission-responsive neurons reported by Lao-Rodríguez

et al. (2023), as such comparisons are not directly meaningful due to fundamental differences in experimental design and recording techniques. Our study focuses on quantitatively characterizing the probability encoding of negative prediction error by PEONs, using varying tone probabilities. Lao-Rodríguez et al., in contrast, used a fixed probability for the standard tone. Additionally, our use of Neuropixels probes allows for simultaneous, unbiased recording from a large population of neurons, whereas previous studies often involved recording from, and potentially selecting, neurons one by one. These differences in approach make direct numerical comparisons of neuron percentages inappropriate.

Regarding Figure 5 (now Figure 2E), we have updated the figure to show the laminar distribution of PEONs as percentages of the total recorded population in each layer.

2.7. Fig 6 C. How are non-selective PEONs identified if, by definition, a PEON has a response correlated with the probability of occurrence of a specific tone?

We thank the reviewer for raising this point. It is important to clarify that neurons classified as PEONs, whether 'selective' or 'non-selective,' exhibit probability encoding for their O_P tone. This is a prerequisite for PEON classification, as detailed in the Methods. The 'non-selective' designation simply indicates that these neurons also show a significant omission response to their O_{NP} tone, in addition to the required response to the O_P tone. Thus, 'non-selectivity' refers to the breadth of the omission response across tones, not to the absence of probability encoding for the preferred tone. The distinction between 'selective' and 'non-selective' PEONs, and the criteria for PEON classification, are clarified in the Results section:

> "To quantify this selectivity across the population, we examined how many PEONs exhibited significant omission responses under different conditions. Out of 123 $PEON_{ODD}$, 90 demonstrated significant O_P -omission responses in EVEN trials (with an additional 5 showing significant responses only to O_{NP} omissions). Of these 90 neurons, 59 (66%) were selective for O_P omissions, whereas 31 (34%) were non-selective, exhibiting significant omission responses for both tones (Figure 4C, left). To further characterize the omission-selective group, we examined their responses to tone presentations to determine whether they responded exclusively to one tone or to both (Figure 4C, right), 41 (or 69%) exhibited significant responses to both the O_P and O_{NP} tones across experimental conditions (see details in Methods). Additionally, 3 PEONs responded exclusively to the O_P tone, 9 solely to the O_{NP} tone, and 6 did not significantly respond to either tone." (page 13 line 285 - page 14 line 295)

We further acknowledge that the split-half analysis now used for PEON identification introduces some additional complexity. A neuron classified as a PEON based on one half of the trials (e.g., ODD trials) might not show statistically significant probability encoding in the independent other half of the trials (e.g., even trials), due to random variability in neuronal responses. However, the core defining characteristic – probability encoding in the training set – is still present.

2.8. How are single units defined?

We thank the reviewer for this question. In the revised manuscript, we have included a detailed description of the criteria used to define single units in the Methods section:

> " Spike sorting was conducted using Kilosort 3 [63], and further manual curation was performed with Phy software (https://phy.readthedocs.io/en/latest/). This process

> involved removing artifacts, merging duplicate clusters, and discarding units with insufficient refractory periods, intermingled waveforms, or unstable firing patterns. Only well-isolated neurons meeting strict inclusion criteria were retained for analysis. The cortical depth of the recording site, estimated via CSD analysis, had to be within 0 µm to +1800 µm from the pial surface. The lower bound of 1800 µm was chosen to confine recordings to the auditory cortex, preventing the inclusion of neurons from subcortical areas or unintended regions. Additionally, neurons had to fire at least 200 spikes per session to ensure sufficient statistical power, while those exhibiting strong drift in firing rate or waveform shape were excluded to maintain stationarity." (page 33 line 778 - page 34 line 788)

2.9. It is probably misleading to use the term "auditory responsive neurons", for neurons responding to tone and or to the omission l. 120, when later in the manuscript "auditory" and "ORN" appear to be separate populations (e.g. Figure 5).

We thank the reviewer for pointing out the potential for confusion caused by the term 'auditory responsive neurons.' We agree that this terminology was misleading, given the subsequent classifications in the original manuscript. In the revised manuscript, we have removed the term 'auditory responsive neurons' and any pre-selection based on general auditory responsiveness. We now identify PEONs directly from the *entire* recorded population, using a split-half analysis, as detailed in the Methods and our response to Reviewer 2.1.

2.10 L. 974 "gray shading" should probably read "red shading"

We thank the reviewer for their careful attention to detail. We have updated the figure legends to further clarify the different elements.

> "The raster plot shows spikes (black dots) with time (ms) on the x-axis and trials on the y-axis. The PSTH displays firing rate over time, with tone stimuli (blue shading) and omissions (gray shading). The black line represents the firing rate, the red line shows omission responses, and the green line indicates the omission baseline. " (page 50 line 1206 - page 51 line 1210)

11. Some introduced abbreviations are redundant e.g. CSD is introduced twice but never used alone as abbreviation.

We thank the reviewer for pointing out the redundant introduction of certain abbreviations (e.g., "CSD"). In the revised manuscript, we have streamlined our use of abbreviations so that each is introduced only once at first use and then used consistently throughout the text without redefinition. This improves clarity and reduces potential confusion.

2.11 Are tone responses e.g. shown in Figure 6 B only responses to the first tone presentation? More details on how tone responses are calculated would be useful.

We appreciate the reviewer's request for clarification regarding how tone responses are calculated in Figure 6B (now Figure 4B). In the revised manuscript, we have added a subsection to the Methods section that provides a detailed explanation of how tone responses were quantified:

> "To ensure that neuronal responses across different tone probability conditions were not biased by differences in statistical power due to varying trial counts, we implemented a standardized subsampling procedure. For each neuron, within each

condition, we limited the analysis of tones to 50 trials per stimulus type to prevent conditions with more trials from exerting disproportionate influence. If more than 50 trials were available, we selected every k-th trial, where k was chosen to achieve 50 trials (e.g., for 100 trials, k = 2; for 200 trials, k = 4, etc.). This ensured that all conditions were analyzed with equal statistical power.

For analyses comparing tone responses with the split-half approach used for PEON identification and characterization (see "PEON Identification" section), an additional subsampling step was performed. After the initial reduction to 50 trials per condition, the remaining trials were further divided into ODD and EVEN subsets, with each subset matched to the corresponding half used for omission responses. "(page 37 line 880 - page 38 line 892)

Regarding Figure 4B, it is important to clarify that tone responses shown in this figure do not exclusively reflect responses to the first tone presentation. Instead, this panel illustrates single-neuron raster plots and PSTHs for omission responses (left column), responses to the O_P tone (middle), and responses to the O_{NP} tone (right), across different probability conditions. Each condition contains 25 subsampled trials, ensuring equalized trial counts across omission and tone response conditions. This approach allows for a balanced and interpretable comparison while maintaining consistency across probability conditions.

2.12. Figure 3 Additional details on the specific sequences used for these plots would be helpful.

We appreciate the reviewer's request for additional details regarding the specific sequences used in Figure 3. In response to a comment from Reviewer 1, we revised our analysis approach to ensure a more rigorous and interpretable assessment of omission response buildup. As part of this revision, we have now explicitly described the sequence structure in both the Results and Methods sections.

Specifically, we now clarify that omission responses were extracted from conditions where the O_P tone was presented as a standard (75%, 85%, 90%, and 95% O_P tone probability) and expected to yield the strongest response. These responses were then grouped by sequence position, allowing us to assess how omission responses evolved over time. To quantify this buildup, we fitted a logistic growth function to the mean omission response across sequence positions, capturing its saturating increase over trials. Additionally, to control for non-specific drift, we analyzed responses to standard tones in the first 50 trials and fitted them with an exponential decay model.

These methodological refinements are now detailed explicitly in the revised Methods:

"For each PEON and each repetition, omission responses were extracted from the four conditions in which the OP tone was presented as the standard (i.e., 75%, 85%, 90%, and 95% OP tone). The omission responses were binned based on their sequence position, and for each bin the mean firing rate and standard error were computed, yielding a two-dimensional response matrix that captures the temporal evolution of omission responses.

The mean omission response was then modeled with a logistic growth function using MATLAB's nlinfit function. The model was defined as: $FR(n) = a / [1 + \exp(-b \times (n - c))]$, where n represents the bin center corresponding to the sequence position, and a,

b, and c are free parameters estimated from the data. The goodness-of-fit was assessed by calculating the R-squared value based on the residual and total sums of squares." (page 38 lines 895-905)

Also in the revised Results:

"To confirm that PEONs encode negative prediction errors, it is essential to show that their responses to omissions increase over time as predictions are gradually formed, reflecting a growing discrepancy between the predicted and actual outcomes. We analyzed $PEON_{ODD}$ from EVEN trials and focused on omissions of the O_P tone in sequences where it was the standard (75%, 85%, 90%, and 95%), and therefore expected to evoke the largest omission responses. We grouped these responses by their sequence position and computed the mean firing rate (± standard error) for each bin. This grouping tests whether the omission response increases as more tones precede the omission, which is the expected pattern if these neurons are indeed signaling a negative prediction error that strengthens with increasing prediction.

Our analysis revealed a notable pattern: the magnitude of the PEONs response to these omissions progressively increased throughout the sequence, achieving a steady state or plateau after approximately 400 standards were presented (Figure 3A). The dynamics of the trial-by-trial omission response were fitted by a logistic growth curve, expressed as:

$$FR(n) = \frac{4.95}{1 + e^{-0.012*(n-223.28)}}$$

where $FR(n)$ represents the firing rate as a function of n, and n is the bin center corresponding to the sequence position of the omissions (i.e., the number of tones presented preceding each omission). The logistic function depicts a saturating increase in response magnitude. Here, an R^2 value of 0.51 indicates the model's moderate success in capturing the variability in responses." (page 11 lines 218-237)

These additions clarify the exact sequence structure, how omission responses were grouped and analyzed, and the rationale for our modeling approach. We thank the reviewer for prompting this clarification, as we believe it improves the transparency and rigor of our analysis.

2.13 L. 383 - l 389 Rationale behind thalamus experiment and conclusions should be explained in more details.

We thank the reviewer for requesting additional clarification about our thalamus experiment. The primary rationale for this experiment was to determine whether the omission responses we observed in cortex might also be present at earlier stages of the auditory pathway, specifically in the thalamus. This would help us understand whether prediction error computation begins subcortically or emerges at the cortical level.

In our preliminary recordings from thalamic neurons, we did not observe robust omission responses. However, the sample size was too limited to include as a formal figure in the paper or to draw definitive conclusions. Based on these preliminary observations, we decided to focus our investigation on cortical mechanisms of prediction error computation. While the thalamic recordings were insufficient for an analysis of omission responses, the tone response properties we observed were consistent with well-established findings in the literature (e.g., Parras et al. 2017; Malmierca et al. 2015). We used only these tone response characteristics

to inform the sensory adaptation parameters in our model (*S_strength*), allowing us to test whether basic adaptation effects alone could account for our cortical observations). As shown in Supplementary Figure S6, our analysis confirmed that the model with lateral PE+ to PE- connections better matched our experimental results than adaptation alone. A full characterization of potential thalamic contributions to predictive processing would require a dedicated study, which is beyond the scope of the current paper.

2.14 Inconsistent number of units used for analysis through the paper: e.g. Table 1 and Table 2 (n= 859 vs n= 751); number of identified PEONs: l. 188 we identified 143 PEONs, l. 226 113 out of 139 PEONs, Figure 9 again 143 PEONs.

We thank the reviewer for their careful attention to detail. These discrepancies are no longer relevant following our revised analyses, as we have updated the analysis and restructured the reporting of unit counts to ensure consistency. All references to neuron counts have been carefully reviewed and corrected, and we have verified that the numbers presented throughout the manuscript now accurately reflect the final analyses.

2.15 Fig 8 What do the three columns in panels D and E represent? This information should be added to the figure. In panel D, what happened to the A response in columns 1 and 3 in the final spike rate graph?

We thank the reviewer for pointing out this lack of clarity in Figure 8 (now Figure 6). The three columns in panels D and E represent different responses of the PEON in Stream 2 under a probability condition of 90% A, 5% B, and 5% O. Specifically, the three columns correspond to neural responses to Tone A (left), Tone B (middle), and omissions (right). We have now added this information explicitly in the figure legend.

Regarding the reviewer's question about panel D, the apparent discrepancy in the A response in columns 1 and 3 is due to the way the spike rasters and peri-stimulus time histograms (PSTHs) are constructed. The rasters display all 500 trials, but since Tone B appears in only 5% of trials while Tone A appears in 90%, this leads to fewer visible spikes for B, despite its stronger per-trial response. The bottom panel, which shows firing rate (spikes/s) rather than total spike count, reveals that B elicits a more robust response when it does occur. This discrepancy is a direct consequence of the lateral interactions in the model, where unexpected stimuli (such as B in this condition) drive stronger responses than expected stimuli (A). We have now clarified this effect in the figure legend

Reviewer #3

In Yaron et al, the authors recorded single-unit activity in the auditory cortex of urethane-anaesthetized rats during an omission paradigm with varying tone probabilities. The aim of this study is to further understand the responses of negative prediction-error neurons. To achieve this aim, they combined electrophysiological recordings with computational modelling.
I believe this study is important as it provides further insight into the omission responses of auditory system neurons. The authors implement a paradigm, which reveals an asymmetry in error signalling and present a model that could explain this phenomena.

We thank the reviewer for their positive assessment and constructive feedback, which have helped improve the clarity and accuracy of our manuscript.

3.1 Authors say that they have recorded 857 auditory neurons out of 1489. From there, in the manuscript (lines 138 and 188) they assert to have 411 ORNs. In figure 2 instead, they assert to have 835 auditory neurons and 404 ORNs. Please double check these numbers and correct or clarify. Check also non-ORNs numbers.

We thank the reviewer for their careful attention to detail. We will double check to avoid these mistakes in our current reanalysis.

3.2 Figure 5. Correct the word 'neruons'. It should be 'neurons'.

We appreciate the reviewer's attention to detail. We have carefully reviewed the text and figures to ensure that all typographical errors, including this one, have been corrected throughout the manuscript. Thank you for your careful reading.

3.3 Figure 5. Personally, I think this figure could be improved from a visual point of view and help the reader to understand it better. In the figure legend, authors mention supragranular (0-600), granular (600-900) and infragranular (900-1400) layers. On the contrary, horizontal dotted lines seem to be labelling different divisions. Please check and correct accordingly. Also, include zero at the top of vertical axis. I also recommend place the little markers for depth and neurons outside the axis for a better visualization.

We appreciate the reviewer's suggestion to improve the clarity of Figure 5 (now 2E). The horizontal dotted lines have been realigned to accurately reflect the supragranular (0–600 µm), granular (600–900 µm), and infragranular (900–1400 µm) boundaries as described in the legend. A zero marker has been added at the top of the vertical axis, and the small markers for neuron counts have been moved outside the axis to reduce visual clutter. These adjustments ensure a clearer depiction of the laminar distribution of neurons.

3.4 Along the manuscript, authors refers to the 150ms between sounds as ISI or SOA. I would like to clarify this:

ISI: Inter-stimulus interval (from the offset of the first stimuli to the onset of the second stimuli).

SOA: Stimulus Onset Asynchrony (from the onset of the first stimuli to the onset of the second stimuli) which includes the stimulus length.

Looking Figure 6 carefully I assume that authors should refer to the 150ms as SOA. In addition, it should be corrected in Figure 7B because the arrow is labelling which is known as SOA.

We appreciate the reviewer's clarification regarding the distinction between ISI and SOA. To ensure consistency and accuracy, we have now revised the manuscript to exclusively use "SOA" when referring to the 150 ms interval. Additionally, we have corrected Figure 7B to ensure the labeling aligns with the correct definition of SOA.
