# Supplementary material for: Auditory cortex neurons that encode negative prediction errors respond to omissions of sounds in a predictable sequence
Source: PLoS Biol. 2025 Jun 18;23(6):e3003242. doi: 10.1371/journal.pbio.3003242 (PMC12212881; doi:10.1371/journal.pbio.3003242)
Supplement: Attachment — Submitted filename: Response to reviewers.pdf [file pbio.3003242.s017.pdf]

REVIEWER REPORTS: PBIOLOGY-D-25-00849R1

Review of "Probability Encoding Omission Neurons in Auditory Cortex Signal Negative Prediction Error"

Reviewer #2

It is commendable that the authors made a serious effort to address our previous concerns.

We sincerely thank Reviewer #2 for their continued careful evaluation of our manuscript and for recognizing our efforts to address the prior concerns. We appreciate the opportunity to clarify the remaining points regarding the implementation of the split-half analysis and the presentation of the corresponding results.

However, regarding the main concern of double dipping, we are not sure this is fully addressed. In their response the authors state that "Trials were divided into two independent subsets—odd and even trials—where one subset was used to classify PEONs and the other was used to quantify omission responses. This prevents circularity by ensuring that selection and characterization are independent." - this would be correct. In the paper, however, it is less clear whether this is implemented systematically. E.g. (line 178): "These findings demonstrate the consistency of the PEON population regardless of which trial subset was used." If this is based on figure 2C, this conclusion is not warranted as the authors would need to show that the PEONodd and PEONeven are the same population of neurons (or at least that the overlap is above chance). In Figure 2D, it appears implemented correctly (but something is amiss with the statistics - should p=1.6x10^27 read p=1.6x10^-27 - and even that does not appear to match the data shown?). The authors should recheck their analysis to confirm that they do not use comparisons of the variety shown in Figure 2C (and fix, or remove Figure 2C).

We thank the reviewer for highlighting the concern regarding potential circularity in our classification and analysis procedures. We agree that clarity on this point is essential and appreciate the opportunity to address it directly. As the reviewer correctly noted, our intended approach was to avoid circularity by selecting neurons based on one subset of trials (e.g., ODD) and characterizing them using the complementary subset (e.g., EVEN). This split-half methodology was systematically implemented in all key analyses involving PEONs, including Figures 2, 3, and 4, to ensure that selection and evaluation were performed on fully independent data. Supplementary Figures S2–S5 present the corresponding results from $PEON_{EVEN}$ and $PEON_{ALL}$, reinforcing the robustness of the findings across trial splits. We have now clarified this point in the main text with the

following sentence:

> “All PEONs were identified using only one half (e.g., ODD) and subsequently tested on the complementary half (e.g., EVEN), ensuring that selection and evaluation were fully independent.” (page 9 lines 162-164)

Regarding Figure 2C and the associated statement in the Results (line 178), we acknowledge the reviewer’s concern that the figure might be interpreted as implying that PEON_{ODD} and $\text{PEON}_{\text{EVEN}}$ reflect the same population of neurons. That was not our intent. Our goal was to show that the proportion of neurons classified as PEONs remained consistent across trial subsets, indicating the robustness of the classification procedure itself. To avoid ambiguity, we have revised Figure 2C in the updated manuscript to display only the results from the ODD trial classification (PEON_{ODD}). The analogous results for $\text{PEON}_{\text{EVEN}}$ and PEON_{ALL} remain available in Supplementary Figure S2, as in the previous version.

Furthermore, to directly address the reviewer’s point regarding population consistency, we quantified the overlap between PEONs identified independently using the ODD and EVEN trial subsets. This analysis revealed a substantial and statistically significant intersection: 61 neurons were classified as PEONs in both subsets. Given the total number of neurons classified in each set, the expected overlap by chance was 18 neurons, and a hypergeometric test confirmed that the observed overlap was highly significant ($p \ll 0.001$). This result provides strong quantitative support for the consistency of the identified PEON population across trial splits, and further validates the robustness of our classification approach.

We have revised the manuscript text accordingly to clarify our intended interpretation:

> “Out of a total of 990 neurons, we identified 123 (13%) PEON_{ODD}, 60 neurons (6%) showed correlation with probability but no omission response, and 807 neurons (82%) showed no correlation (Figure 2C, top). On the other hand, when using the EVEN trials, 145 neurons were classified as $\text{PEON}_{\text{EVEN}}$, 69 showed correlation but no response, and 776 showed no correlation. Although the precise numbers varied slightly between subsets, the classification proportions were broadly similar, supporting the robustness of the procedure. To assess consistency more directly, we quantified the overlap between the two independently identified PEON subsets. A total of 61 neurons were classified as PEONs in both ODD and EVEN trial sets, significantly exceeding the overlap expected by chance (expected: 18 neurons; hypergeometric test, $p \ll 0.001$). This result supports the stability of the classification approach, even when applied to trial-split subsets with reduced statistical power. For comparison, when using all trials without splitting, 200 neurons were classified as PEON_{ALL}, 70 showed correlation but no response, and 720 showed no

correlation. Pie charts for both $PEON_{EVEN}$ and $PEON_{ALL}$ classifications are shown in Supplementary Figure S2." (page 9 lines 177-190)

Regarding Figure 2D, we confirm that the split-half analysis was correctly implemented, with PEONs identified using ODD trials ($PEON_{ODD}$) and characterized using EVEN trials. The reviewer is correct that the p-value in the original figure caption contained a typographical error in the exponent. The correct value, as stated in the main text, is $p = 1.6 \times 10^{-27}$, based on Spearman's rho = 0.34 across 984 data points (123 neurons × 8 conditions). This has been corrected in the revised figure caption.

As for the reviewer's concern that the statistical result may not match the visual trend, we note that while the relationship is modest ($\rho = 0.34$), it is highly significant and directionally consistent with the predicted link between omission response magnitude and O_P tone probability.

Reviewer #3

The authors have addressed all my comments.

We thank Reviewer #3 for their positive assessment and confirmation that their previous comments have been satisfactorily addressed.
